# Multi‐Omics Insights Into the Mechanisms of Early Muscle Fiber Difference and Transformation Between Lean‐Type and Chinese Indigenous Pigs

**DOI:** 10.1002/advs.202523959

**Published:** 2026-04-17

**Authors:** Shuailong Zheng, Hainan Wu, Min Liu, Kunpeng Wu, Shuntao Huang, Zulfiqar Ahmed, Lenox Pius, Mengjin Zhu, Dequan Xu

**Affiliations:** ^1^ Key Laboratory of Swine Genetics and Breeding of the Ministry of Agriculture and Rural Affairs Huazhong Agricultural University Wuhan China; ^2^ Key Laboratory of Agricultural Animal Genetics Breeding and Reproduction of the Ministry of Education Huazhong Agricultural University Wuhan China; ^3^ Colleges of Animal Science & Technology Huazhong Agricultural University Wuhan China; ^4^ Colleges of Veterinary Medicine Huazhong Agricultural University Wuhan China

**Keywords:** chromatin architecture, multi‐omics integration, muscle fiber, pig, PPP3CB, super‐enhancer

## Abstract

Skeletal muscle fiber composition is a key determinant of meat quality and metabolic traits. The early postnatal period constitutes a primary window for muscle fiber transformation. In this study, distinct muscle fiber composition is analyzed by histological and molecular characterization of longissimus dorsi muscle of 2‐week‐old lean‐type and Chinese indigenous pigs. Multi‐omics analyses reveal divergence in chromatin states, enhancer landscapes, and promoter activity between breeds. Lean‐type pigs show increased chromatin accessibility and enhancer activation at genes involved in oxidative metabolism and myogenesis, whereas Chinese indigenous pigs exhibit enriched regulatory features at glycolytic and biosynthetic loci. Breed‐specific SNPs and indels are enriched in a subset of enhancers and potentially influence transcriptional regulation. Higher‐order genome architecture further contributes to transcriptional divergence through A/B compartment switching and topologically associating domain boundary remodeling. Promoter–enhancer interaction mapping reveals breed‐specific cis‐regulatory networks, and transcriptional output correlates with enhancer density. Functional validation identifies a super‐enhancer upstream of *PPP3CB* that recruits MEF2C to activate oxidative fiber programs. In vitro and in vivo perturbation assays confirm that the *PPP3CB–*MEF2C feedback loop governs muscle fiber‐type specification. Collectively, these findings delineate the epigenomic and 3D genomic architecture underlying early muscle fiber characteristics and provide mechanistic insights relevant to improving meat quality.

## Graphical abstract

Multi‐omics analyses uncover breed‐specific cis‐regulatory landscapes and higher‐order chromatin architectural differences that underlie early postnatal muscle fiber divergence in pigs. A super‐enhancer upstream of *PPP3CB* recruits MEF2C to activate *PPP3CB* transcription, while the *PPP3CB*–MEF2C positive feedback loop promotes oxidative muscle fiber identity, providing mechanistic insights into muscle fiber transformation and improvement of meat quality.

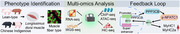

## 1 Introduction

Skeletal muscle is a heterogeneous tissue composed of multiple fiber types with distinct metabolic and contractile properties that support locomotion, postural control, systemic metabolic homeostasis, and meat quality [1]. Pigs (*Sus scrofa*) are a major livestock species for meat production and a physiologically relevant model for human muscle biology because of their comparable anatomy, metabolism, and genomic features [2]. Pig skeletal muscle comprises oxidative fibers (type I/MyHC1 and type IIa/MyHC2a), intermediate fibers (type IIx/MyHC2x), and glycolytic fibers (type IIb/MyHC2b), distinguished by myosin heavy chain (MyHC) isoform expression and mitochondrial content and governed by coordinated transcriptional programs [3]. Muscle fiber composition undergoes dynamic remodeling during porcine growth. Due to inherent growth characteristics, the timing of muscle development and fiber transformation in Chinese indigenous pigs differs from that in lean‐type pigs [4]. This temporal difference provides a unique perspective for studying the molecular mechanisms underlying muscle fiber differences and transformation.

Previous studies have shown that muscle‐fiber differentiation is regulated by a complex network of signaling pathways, involving the coordinated action of various transcription factors, such as wingless‐integrated (WNT) [5], phosphoinositide 3‐kinase (PI3K)–Akt [6], and mitogen‐activated protein kinase (MAPK) signaling pathways [7]. These signaling pathways work together to regulate the transition of muscle fiber types. Moreover, metabolic reprogramming plays an important role in muscle fiber type transformation. Under the regulation of nutritional status and exercise load, muscle cells adapt to different functional demands by adjusting their metabolic pathways, thereby promoting the transformation of muscle fiber types [8, 9]. Recent research in muscle development has shown that RNA‐binding proteins regulate the proportion of slow muscle fibers and mitochondrial function through liquid–liquid phase separation [10].

Despite the recognized significance of muscle fiber composition, the regulatory mechanisms governing its early establishment remain incompletely understood, particularly from a chromatin biology perspective [11]. Epigenetic alterations, including chromatin accessibility [12], histone acetylation and methylation [13], and higher‐order genome architecture [14], are crucial for producing lineage‐specific gene expression profiles without altering the underlying DNA sequence. The three‐dimensional (3D) genome is organized into A/B compartments [15], topologically associating domains (TADs) [16], and promoter–enhancer interactions (PEIs) [17, 18], which together provide a structural framework for transcriptional regulation and cellular identity [19]. Advancements in multi‐omics profiling techniques have facilitated a deeper understanding of these regulatory layers. Recent research has revealed the spatial conformation of skeletal muscle chromatin in pigs of different metabolic types and its differential regulation during muscle fiber transformation [20]. However, there is a notable gap in research examining differences in muscle fiber composition and fiber‐type transformation among pig breeds, particularly in relation to 3D genomic architecture, chromatin accessibility, histone modification patterns, and transcriptional output.

The transformation of muscle fiber types is of significant importance in postnatal muscle development, particularly during the early postnatal period of pigs (0–14 days), a primary period for muscle remodeling [21, 22]. Accordingly, porcine postnatal day 14 (P14d), that represents an early developmental window, is well suited for capturing breed‐associated divergence in myofiber characteristics. In this study, a comprehensive multi‐layered regulatory atlas of the *longissimus dorsi* (LD) muscle was created by integrating RNA sequencing (RNA‐seq), Assay for Transposase‐Accessible Chromatin with high‐throughput sequencing (ATAC‐seq), Chromatin Immunoprecipitation sequencing (ChIP‐seq) targeting histone H3 lysine 27 acetylation (H3K27ac) and histone H3 lysine 4 trimethylation (H3K4me3), whole‐genome sequencing (WGS), and in situ High‐throughput chromosome conformation capture (Hi‐C) datasets from 2‐week‐old lean‐type and Chinese indigenous pigs. Genome‐wide active regulatory elements, variants, chromatin states, and higher‐order structures were delineated and correlated with differential gene expression related to fiber‐type‐specific characteristics. Furthermore, our findings indicated significant variation in chromatin compartmentalization, TAD dynamics, and PEIs across breeds, suggesting that structural genomic remodeling plays an important role in muscle fiber composition. Notably, we identified a lean‐type‐specific super‐enhancer–promoter loop that regulates protein phosphatase 3 catalytic subunit beta (*PPP3CB*) through myocyte enhancer factor 2C (MEF2C) recruitment, thereby establishing a molecular circuit that promotes oxidative muscle fiber identity. Together, these results provide mechanistic insights and regulatory targets that may inform genetic and epigenetic strategies to improve meat quality.

## 2 Results

### 2.1 Histological and Molecular Analyses Indicate a More Oxidative Muscle Fiber in the LD Muscle of Lean‐Type Pigs Than in Chinese Indigenous Pigs at Two Weeks of Age

**FIGURE 1:**
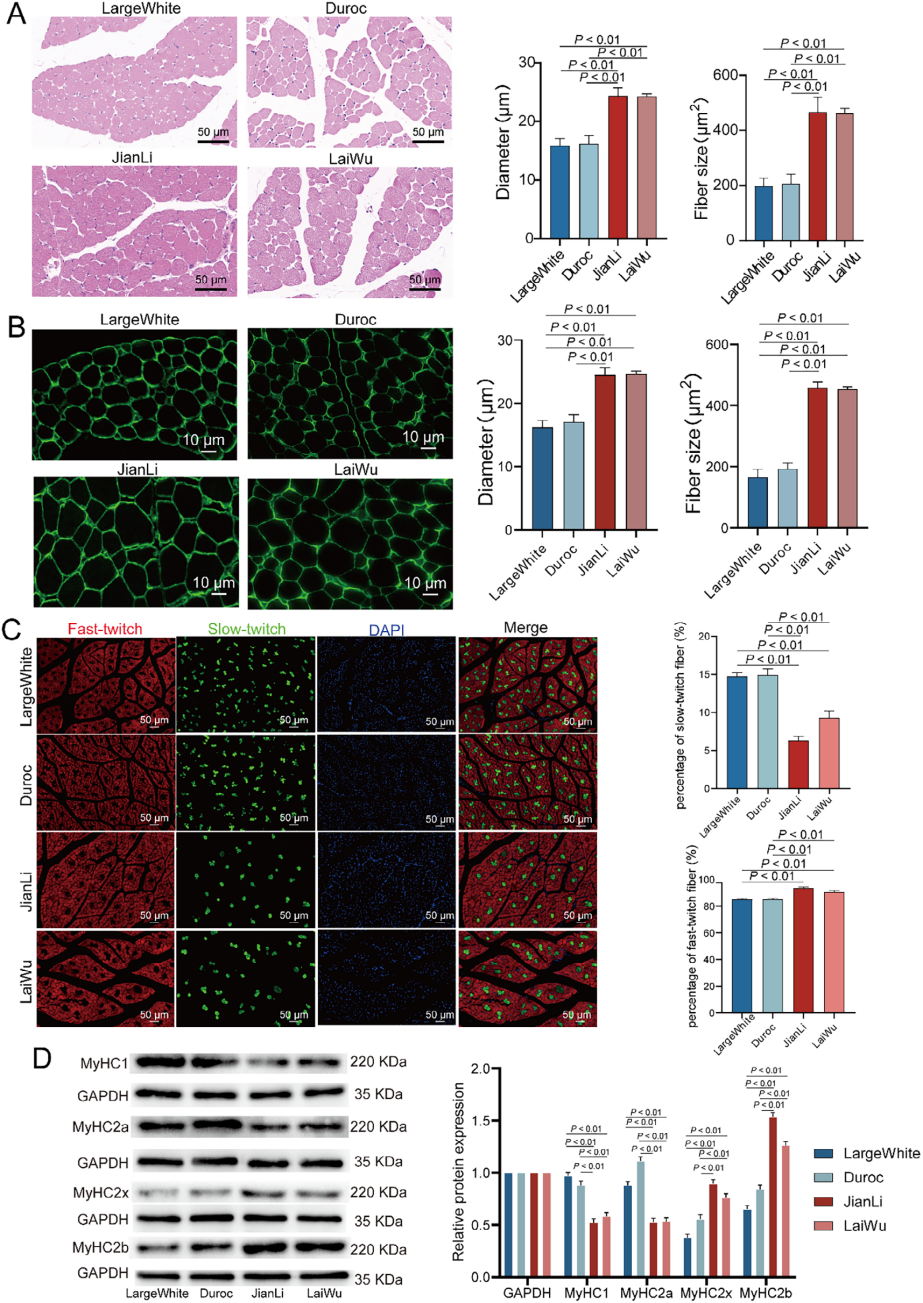
Histological and molecular analysis of the longissimus dorsi (LD) muscle in 2‐week‐old lean‐type and Chinese indigenous pigs. (A) Representative H&E‐stained sections of LD muscle from 2‐week‐old pigs (LargeWhite, Duroc, JianLi, and LaiWu; n = 3 per breed). Scale bar, 50 µm. (B) Representative dystrophin immunofluorescence images outlining myofiber boundaries (n = 3 per breed). Scale bar, 10 µm. (C) Representative immunofluorescence staining for fast‐ and slow‐twitch fiber markers in LD (n = 3 per breed). Scale bar, 50 µm. (D) Western blot analysis and densitometric quantification of MyHC isoforms (MyHC1, MyHC2a, MyHC2b, and MyHC2x) in LD muscle (n = 3 per breed). Band intensities were normalized to GAPDH. For quantification in (A–C), ≥150 myofibers were analyzed per pig, and per‐animal means were used for statistical analysis (biological replicate = pig). Data are shown as mean ± SD. *p* values were calculated using two‐sided unpaired Student's t‐tests for the following pairwise comparisons: LargeWhite vs JianLi, LargeWhite vs LaiWu, Duroc vs JianLi, and Duroc vs LaiWu.

Histological analyses (H&E staining and dystrophin immunofluorescence) were performed on the LD muscle of 2‐week‐old pigs from four breeds, including lean‐type breeds (LargeWhite and Duroc) and Chinese indigenous breeds (JianLi and LaiWu). H&E‐stained sections, lean‐type pigs exhibited significantly smaller myofibers than Chinese indigenous pigs, with reduced average diameter (16.03 ± 1.22 µm vs. 24.31 ± 0.98 µm; *p* < 0.01) and cross‐sectional area (203.02 ± 30.08 µm^2^ vs. 464.80 ± 36.96 µm^2^; *p* < 0.01) (Figure 1A). Consistent with the H&E findings, dystrophin immunofluorescence staining clearly delineated myofiber boundaries and confirmed the reduced myofiber size in lean‐type pigs, enabling robust segmentation for morphometric quantification (Figure 1B).

Immunofluorescence staining revealed that lean‐type pigs had a significantly higher proportion of slow‐twitch (MyHC1) fibers than Chinese indigenous breeds (14.83% ± 0.58% vs. 7.79% ± 1.78%; *p* < 0.01), although fast‐twitch fibers predominated in both groups (Figure 1C). These findings were corroborated by qPCR and Western blotting. Lean‐type pigs showed higher expression of the oxidative fiber markers MyHC1 and MyHC2a, whereas Chinese indigenous pigs showed higher expression of the glycolytic marker MyHC2b and the intermediate marker MyHC2x (Figure 1D, Figure S1A, Supporting Information). Enzyme activity assays further supported these results. 2‐week‐old lean‐type pigs exhibited higher activities of oxidative enzymes, including succinate dehydrogenase (SDH) and malate dehydrogenase (MDH), and lower activity of the glycolytic enzyme lactate dehydrogenase (LDH) in LD muscle (*p* < 0.05) (Figure S1B, Supporting Information). To summarize breed‐type differences across all measured traits, we applied a linear mixed‐effects model and estimated the adjusted effect of breed type (lean‐type vs. Chinese indigenous) for each indicator. The mixed‐model–estimated breed type effects with 95% confidence intervals are summarized in the forest plot (Figure S1C) and visualized across indicators in the heatmap (Figure S1D). Overall, the mixed‐model results indicated higher oxidative fiber–associated indicators in lean‐type pigs than in Chinese indigenous pigs. Meanwhile, in adulthood, Western blotting and enzyme assays indicated relatively higher glycolytic/intermediate MyHC signatures and glycolytic enzyme activity in LargeWhite pigs than in JianLi pigs (Figure S1E,F, Supporting Information).

In summary, at 2 weeks of age, LD muscle from lean‐type pigs showed a higher proportion of MyHC1‐positive fibers and a more oxidative MyHC isoform profile (higher MyHC1/MyHC2a and lower MyHC2b/MyHC2x) than that from Chinese indigenous pigs.

### 2.2 Dynamic Epigenomic Characteristics Correlate With Gene Expression Variability Affecting Muscle Fiber Composition

**FIGURE 2:**
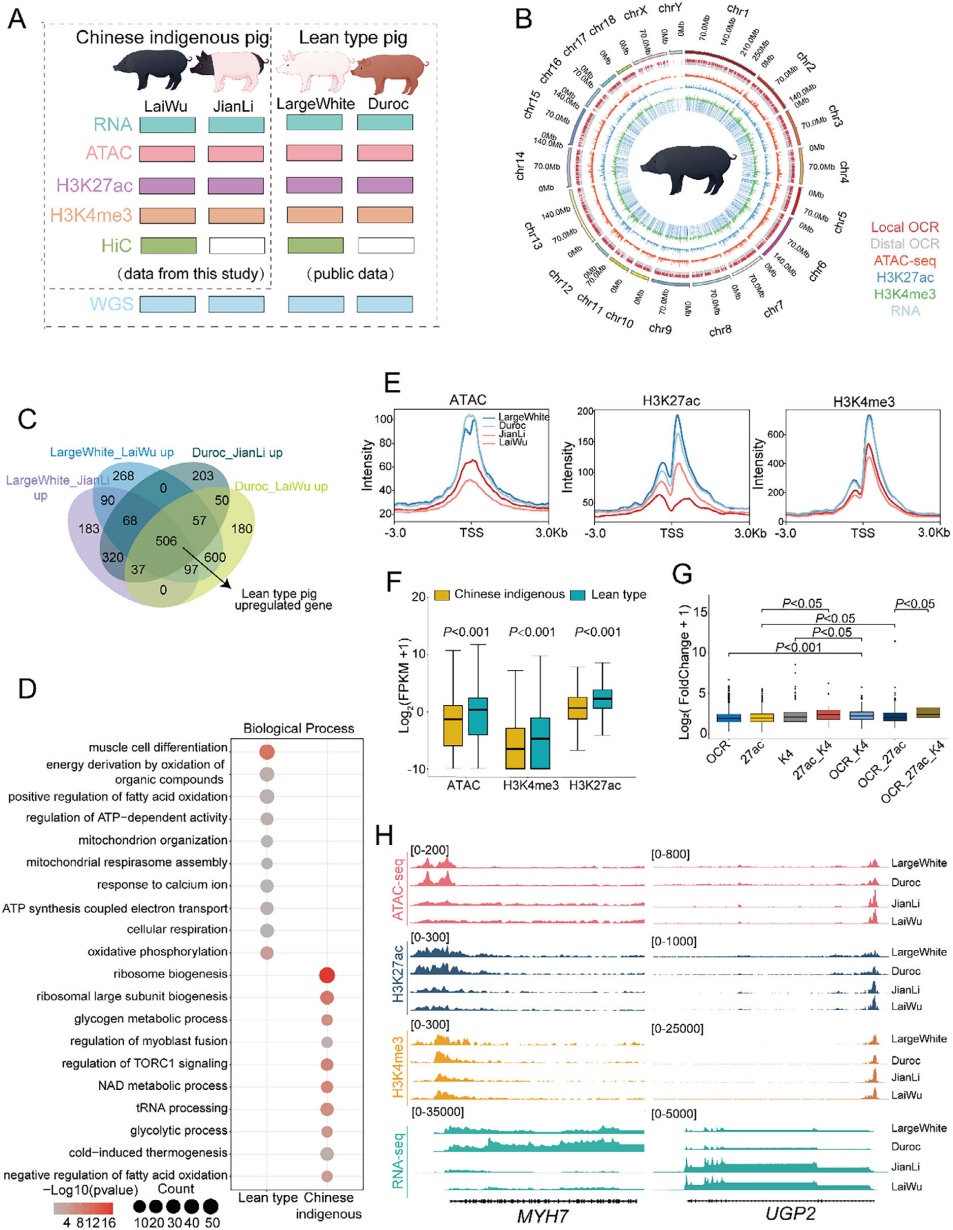
Dynamic OCRs and histone modifications associated with breed‐specific gene expression in lean‐type and Chinese indigenous pigs. (A) Schematic illustration of the experimental workflow. (B) Distribution of LoOCRs, dOCRs, and histone modifications (H3K4me3, H3K27ac) in LaiWu pig. (C) Overlap of genes upregulated in lean‐type breeds across four pairwise comparisons (LargeWhite vs. LaiWu, LargeWhite vs. JianLi, Duroc vs. LaiWu, and Duroc vs. JianLi). (D) GO analysis of biological processes for commonly upregulated genes in indigenous pigs or lean‐type pigs. (E) Average ATAC‐seq, H3K27ac, and H3K4me3 signal intensities within a 3 kb window around the transcription start site (TSS) of genes upregulated in lean‐type pigs. (F) Expression levels of genes enriched in ATAC‐seq, H3K27ac, and H3K4me3 alterations in lean‐type pigs. (G) Fold change in the expression of gene sets enriched in lean‐type pigs, correlated with alterations in single or combination chromatin states. K4 denotes H3K4me3; 27ac denotes H3K27ac. (H) Examples of *MYH7* (upregulated in lean‐type pig) and *UGP2* (upregulated in Chinese indigenous pig), showing expression and chromatin state differences between lean‐type and Chinese indigenous pigs. *p* values were calculated using a two‐sided Wilcoxon rank‐sum test in (F, G).

To elucidate the regulatory mechanisms underlying muscle fiber differences, we generated and analyzed 14 ChIP‐seq, 4 RNA‐seq, 4 ATAC‐seq, and 2 Hi‐C datasets from LD muscle of 2‐week‐old Chinese indigenous pigs. In addition, we incorporated 46 publicly available datasets for integrative comparisons (Figure 2A, Table S1, Supporting Information). Principal component analysis (PCA) and pairwise correlation analyses demonstrated high concordance between biological replicates, supporting data robustness (Figure S2A–G, Supporting Information). ATAC‐seq profiling identified ∼99 702 shared open chromatin regions (OCRs) and a small subset of breed‐specific OCRs, suggesting that most OCRs are conserved across breeds (Figure S3A, Supporting Information). Approximately 65% of OCRs localized to gene bodies or promoter regions (local OCRs; LoOCRs), whereas ∼35% were distal OCRs (dOCRs) (Figure S3B, Table S2, Supporting Information). Within each breed, chromatin accessibility and histone modification signals were enriched at LoOCRs and comparatively weaker at dOCRs (Figure 2B, Figure S3C,D, Supporting Information). Genes associated with LoOCRs generally showed higher expression levels than those linked to dOCRs (Figure S3E, Supporting Information). Motif analysis revealed enrichment of predicted binding motifs for MEF2C and myocyte enhancer factor 2A (MEF2A) in LoOCRs from lean‐type pigs, suggesting breed‐biased transcription factor engagement during fiber‐type remodeling (Figure S4, Supporting Information).

Given the phenotypic differences, we next assessed whether breed divergence in muscle fiber characteristics was accompanied by transcriptomic changes. RNA‐seq analysis identified 506 genes upregulated in lean‐type pigs and 327 genes enriched in Chinese indigenous pigs (*P*adj < 0.05, |log_2_FC| > 1.5) (Figure 2C, Figure S5A, Supporting Information). In 2‐week‐old lean‐type pigs, upregulated genes (e.g., troponin T type 1 (*TNNT1*) [23], myosin heavy chain 7 (*MYH7*) [24], and myosin heavy chain 2 (*MYH2*) [25]) were enriched for terms related to oxidative phosphorylation, cellular respiration, calcium ion responses, and positive regulation of fatty acid oxidation. In contrast, in 2‐week‐old Chinese indigenous pigs, upregulated genes (e.g., phosphofructokinase (*PFKM*) [26], UDP‐glucose pyrophosphorylase 2 (*UGP2*) [27], and hexokinase 2 (*HK2*) [28]) were enriched for terms related to glycolysis, glycogen metabolism, and NAD metabolism (Figure 2D, Figure S5B, Supporting Information). ATAC‐seq, H3K27ac, and H3K4me3 signals were significantly higher around transcription start sites of lean‐type–upregulated and indigenous‐upregulated genes (*p* < 0.01, Wilcoxon test) (Figure 2E, Figure S5C, Supporting Information).

Dynamic chromatin accessibility and histone modifications reflected the differences in gene expression. Next, we used DiffBind to identify differential ATAC‐seq peaks and differential H3K27ac and H3K4me3 peaks between lean‐type and Chinese indigenous pigs, considering both local and distal regions (Figure S5D,E, Supporting Information). Genes associated with OCRs, H3K27ac, and H3K4me3 peaks enriched in lean‐type pigs exhibited significantly higher expression than those in Chinese indigenous pigs (*p* < 0.001, Wilcoxon test) (Figure 2F). Conversely, genes linked to regions enriched for these marks in Chinese indigenous pigs showed higher expression than their counterparts in lean‐type pigs (*p* < 0.001, Wilcoxon test) (Figure S5F, Supporting Information). Notably, genes supported by combinatorial enrichment of chromatin features (OCRs, H3K27ac, and H3K4me3) displayed the largest expression changes compared with genes associated with any single mark (Figure 2G, Figure S5G, Supporting Information), including key myofiber‐related genes such as *MYH7*, calcium/calmodulin dependent protein kinase II alpha (*CAMK2A*) [29], *HK2*, and *UGP2* (Figure 2H, Figure S5H, Supporting Information).

Collectively, these results indicate that breed‐specific chromatin accessibility and activating histone modifications are associated with differential gene expression programs that likely contribute to early muscle fiber composition.

### 2.3 Divergence of Breed‐Specific Regulatory Elements and Genetic Variations Contributing to Muscle Fiber Composition

**FIGURE 3:**
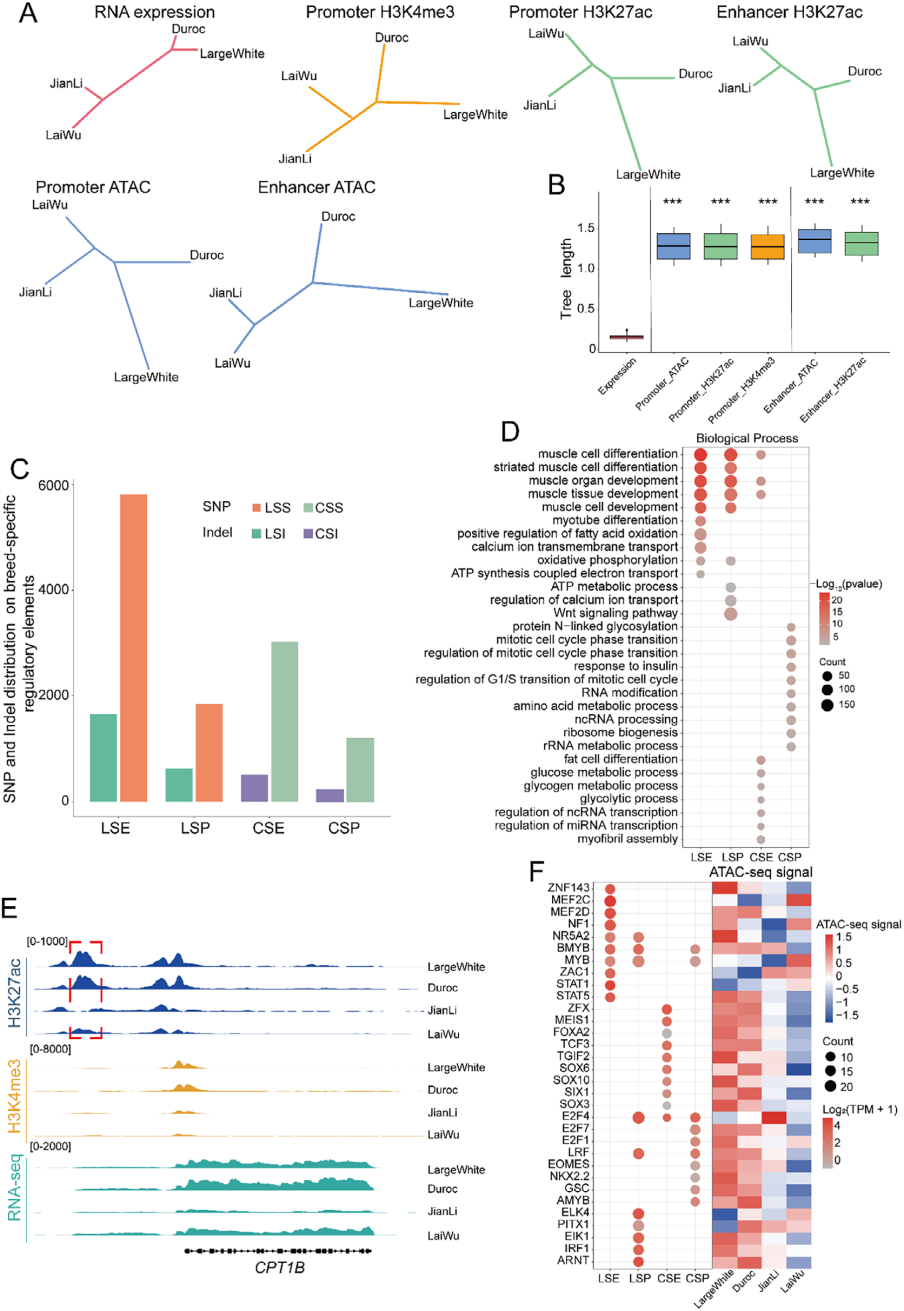
Divergence of CREs, genetic variation, and gene expression in lean‐type and Chinese indigenous pigs. (A) Phylogenetic trees were built using the neighbor‐joining method, showing gene expression, H3K4me3, H3K27ac, and chromatin accessibility based on enhancer and promoter data. (B) Comparison of branch lengths across omics layers using RNA‐seq as the reference; significance was assessed by 1000 bootstrap resamplings (***p < 0.001). (C) Distribution of breed‐specific SNPs and indels within breed‐specific active regulatory elements in Chinese indigenous and lean‐type pigs. LSP, active promoters specific to lean‐type pigs; LSE, active enhancers specific to lean‐type pigs; CSP, active promoters specific to Chinese indigenous pigs; CSE, active enhancers specific to Chinese indigenous pigs. LSS, SNPs specific to lean‐type pigs; LSI, indels specific to lean‐type pigs; CSS, SNPs specific to Chinese indigenous pigs; CSI, indels specific to Chinese indigenous pigs. (D) GO biological process enrichment analysis of genes associated with lean‐type–specific and Chinese indigenous–specific active enhancers and promoters. (E) A lean‐type‐specific active enhancer located upstream of *CPT1B* (red arc). (F) Motif enrichment analysis for lean‐type–specific and Chinese indigenous–specific active enhancers and promoters.

*Cis*‐regulatory elements (CREs) regulate transcription by recruiting sequence‐specific transcription factors [30, 31]. Analysis of H3K4me3 and H3K27ac profiles identified a significantly larger number of active promoters, enhancers, and super‐enhancers in lean‐type pigs than in Chinese indigenous pigs (Figure S6A–C, Supporting Information). By contrast, whole‐genome sequencing (WGS) identified a higher total number of SNPs and indels in Chinese indigenous pigs than in lean‐type pigs (Figure S7A, Supporting Information). Consistent with WGS‐based divergence, phylogenetic trees constructed from RNA‐seq, ATAC‐seq, and ChIP‐seq data also separated lean‐type and Chinese indigenous breeds, supporting coordinated divergence of gene expression and regulatory landscapes (Figure 3A and Figure S7B, Supporting Information). Promoters were more conserved than enhancers, and gene expression was more stable than epigenomic features (Figure 3B).

Active cis‐regulatory elements (CREs) were classified into three groups, including conserved active promoters (ACP) and enhancers (ACE), lean‐type‐specific active promoters (LSP) and enhancers (LSE), and Chinese indigenous–specific active promoters (CSP) and enhancers (CSE). Lean‐type pigs exhibited a markedly higher proportion of breed‐specific active CREs, as 77.4% of their active enhancers and 45.8% of active promoters were specific. In contrast, only 28.4% of active enhancers and 10.9% of active promoters were specific to Chinese indigenous pigs (Figure S6D, Supporting Information). In contrast, we found that Chinese indigenous pigs exhibited a significantly higher proportion of breed‐specific variants, with 77.3% of the specific SNPs (CSS) and 67.8% of the specific indels (CSI). In contrast, only 50.6% of the specific SNPs (LSS) and 49.8% of the specific indels (LSI) were specific to lean‐type pigs (Figure S7C,D, Supporting Information). To examine the genetic basis of this regulatory divergence, we quantified enrichment of group‐specific variants (lean‐type‐specific vs. Chinese indigenous–specific) within the corresponding CRE categories. Both lean‐type‐specific and Chinese indigenous‐specific variants were higher enriched in breed‐specific enhancers (LSE/CSE) than in breed‐specific promoters (LSP/CSP) (Figure 3C). These results highlight enhancers as major contributors to breed‐specific regulatory divergence, given their lower conservation, higher breed specificity, and greater accumulation of putatively functional variants than promoters.

GO enrichment analysis revealed that the specific active enhancers and promoters in 2‐week‐old lean‐type pigs were enriched for processes related to muscle cell differentiation, positive regulation of fatty acid oxidation, calcium ion transport, oxidative phosphorylation, and ATP synthesis (Figure 3D). For example, a lean‐type‐specific enhancer was located upstream of carnitine palmitoyltransferase 1B (*CPT1B*) [32], a key regulator of fatty acid oxidation relevant to oxidative fiber function (Figure 3E). In contrast, Chinese indigenous–specific CRE‐associated genes were enriched for terms related to glycolysis, glucose metabolism, and insulin responses, consistent with a greater contribution of glycolytic and intermediate fibers in these pigs (Figure 3D). For example, a Chinese indigenous–specific enhancer was identified near pyruvate kinase M1/2 (*PKM*) [33], which encodes the terminal rate‐limiting enzyme in glycolysis (Figure S7E, Supporting Information).

Motif enrichment analysis showed that lean‐type‐specific enhancers were enriched for motifs recognized by MEF2C, myocyte enhancer factor 2D (MEF2D) [34], and nuclear receptor subfamily 5 group A member 2 (NR5A2), which have been implicated in oxidative fiber programs. Conversely, Chinese indigenous–specific enhancers were enriched for SRY‐box transcription factor 6 (SOX6), SRY‐box transcription factor 10 (SOX10), and SIX homeobox 1 (SIX1) motifs; SOX6 [35] and SIX1 [36] have been reported to promote slow‐to‐fast fiber‐type transitions in skeletal muscle (Figure 3F). To further evaluate transcription factor engagement, we performed ATAC‐seq footprint analysis. Footprint analysis indicated higher binding scores at MEF2C/MEF2D/NR5A2 motif sites within LSEs in lean‐type pigs and at SIX1/SOX6/SOX10 motif sites within CSEs in Chinese indigenous pigs (Figure S6E, Supporting Information). Collectively, breed‐specific differences in CRE landscapes were associated with divergent muscle fiber composition between lean‐type and Chinese indigenous pigs.

### 2.4 Chromatin Compartments and TAD Dynamics Regulate Transcriptional Rewiring and Muscle Fiber Identity

**FIGURE 4:**
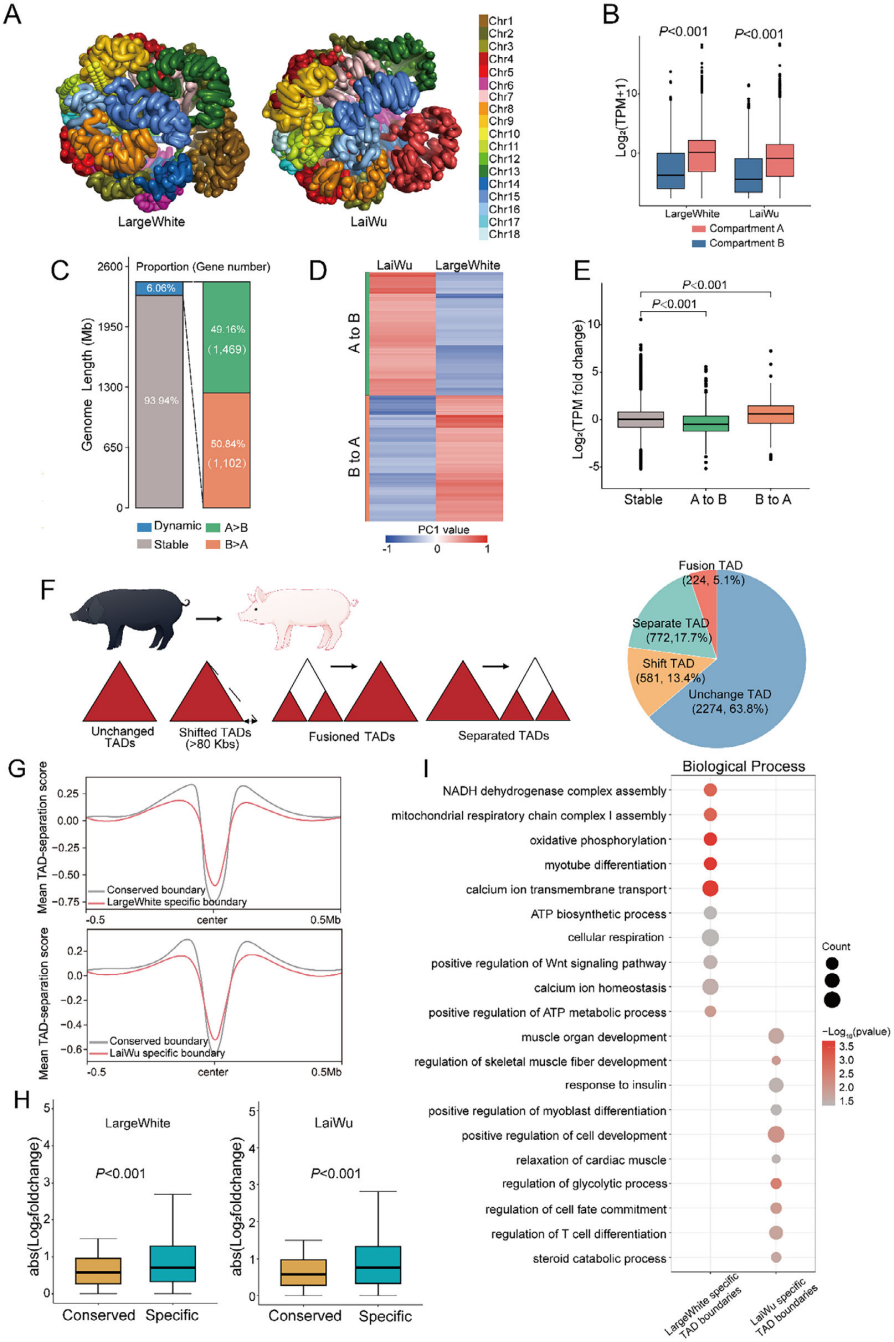
Compartmentalization and TAD dynamics between lean‐type and Chinese indigenous pigs. (A) Simulated 3D genome organization in LargeWhite and LaiWu pigs. (B) Gene expression [Log_2_(TPM + 1)] of genes located in A or B compartments. (C) Genomic lengths and proportions of stable versus dynamic compartments, with dynamic compartments classified by transition type. (D) Heatmap of PC1 values for compartment‐switching regions. (E) Expression levels of differentially expressed genes (DEGs) in compartment‐switching regions versus stable compartments. (F) Changes in TAD boundaries and distribution of boundary categories between breeds. (G) Comparison of mean TAD separation scores between conserved and breed‐specific boundaries. (H) Absolute expression changes for genes located at conserved versus breed‐specific TAD boundaries. (I) GO biological process enrichment analysis of genes associated with breed‐specific TAD boundaries. *p* values were calculated using a two‐sided Wilcoxon rank‐sum test in (B,E,H).

Chromatin interactions between CREs and their target genes are crucial for transcriptional regulation [37, 38]. To explore these interactions, we generated in situ Hi‐C data for a 2‐week‐old LaiWu pig and integrated these with publicly available Hi‐C data for 2‐week‐old LargeWhite pigs to construct chromatin contact maps. Hi‐C yielded ∼1.48 billion valid contacts, including 74.04% cis interactions and ∼47% long‐range interactions, indicating high data quality (Figure S8A–F, Figure S9A, Table S1, Supporting Information). 3D genome modeling further illustrated the spatial segregation of chromosomal regions, consistent with chromosome territories in the pig genome (Figure 4A). Compartment analysis indicated that 49.04% of the genome was assigned to the active A compartment in LargeWhite pigs, compared with 50.74% in LaiWu pigs (Figure 4B, Figure S9B,C, Table S3, Supporting Information). Epigenomic profiling further supported this division, with A compartments displaying higher chromatin accessibility and elevated levels of H3K27ac and H3K4me3 compared to B compartments (Figure S9D, Supporting Information).

Notably, LargeWhite pigs showed significantly higher compartment strength than LaiWu pigs (Figure S9E, Supporting Information). Approximately 6.06% of the genome exhibited compartment switching between the breeds; among switching regions, 49.16% transitioned from A to B (1469 genes) and 50.84% from B to A (1102 genes) (Figure 4C,D, Figure S10A, Supporting Information). For instance, a representative region on chromosome 17 was visualized at higher resolution to integrate PC1‐defined A/B compartment switching with breed‐specific active regulatory elements (CSE/CSP and LSE/LSP), suggesting that compartment switching may influence the formation of breed‐specific active CREs (Figure S10B,C, Supporting Information). These compartment transitions were associated with concordant changes in gene expression (Figure 4E). Specifically, genes located within regions undergoing B‐to‐A compartment switching (LaiWu B to LargeWhite A) were enriched for terms related to calcium ion transmembrane transport, a process linked to oxidative fiber formation (e.g., calcium/calmodulin dependent protein kinase ID (*CAMK1D*) [39]). In contrast, genes transitioning from A to B were enriched for processes such as regulation of glycolysis (e.g., glycerol‐3‐phosphate dehydrogenase 1 (*GPD1*) [40]) (Figure S10D,E, Supporting Information). Consistently, qPCR confirmed differential expression of *CAMK1D* and *GPD1* between breeds (Figure S10F, Supporting Information). These compartment dynamics underscore the spatial and functional reprogramming of the transcriptional landscapes underlying breed‐specific muscle fiber phenotypes.

At higher resolution, TAD analysis identified 5038 boundaries in LaiWu and 4379 in LargeWhite pigs, of which 53.61% were conserved, indicating additional architectural divergence (Figure S11A, Table S3, Supporting Information). In total, 5008 TADs in LargeWhite and 4351 TADs in LaiWu pigs were annotated. Average TAD size was comparable between breeds (∼400 kb) (Figure S11B,C, Table S3, Supporting Information), but boundary insulation strength was lower in LaiWu pigs (Figure S11D,E, Supporting Information). TADs enriched in A compartments tended to be smaller than those in B compartments (Figure S11F, Supporting Information). Notably, 13.4% of the boundaries were shifted, 5.1% were fused, and 17.7% were separated (Figure 4F, Table S3, Supporting Information). These rearrangements from LaiWu to LargeWhite pigs were linked to expression changes, such as increased expression of follistatin (*FST*) [41], which is involved in regulating glycolytic fiber formation, near a fused boundary in 2‐week‐old LaiWu pigs (Figure S11G, Supporting Information).

Epigenomic profiling at TAD boundaries showed a gradual decrease in H3K4me3 signal from the center toward adjacent regions, suggesting that the central region of the TAD boundary may be a gene‐active area (Figure S11H, Supporting Information). Conserved boundaries displayed stronger insulation scores in both breeds (Figure 4G). Genes located at breed‐specific boundaries showed significantly higher expression levels than those at conserved boundaries, supporting a link between boundary remodeling and transcriptional divergence (Figure 4H). Gene Ontology analysis further indicated that LargeWhite‐specific TAD boundaries were enriched in genes (e.g., *NDUFAF4* (NADH:ubiquinone oxidoreductase complex assembly factor 4), *NDUFB3* (NADH:ubiquinone oxidoreductase subunit B3), and *NDUFAF5* (NADH:ubiquinone oxidoreductase complex assembly factor 5)) [42] associated with mitochondrial respiration and calcium ion transport, whereas LaiWu‐specific boundaries favored genes (e.g., *GPD1*, *PFKM*) involved in muscle development, regulation of glycolytic process, and cardiac muscle relaxation (Figure 4I). Consistently, qPCR validated differential expression of representative boundary‐associated genes (Figure S11I, Supporting Information). Motif enrichment and ATAC‐seq footprint analysis suggested differential MEF2C engagement, with higher footprint scores at LargeWhite–specific TAD boundaries (Figure S11J,K, Supporting Information). Additionally, CCCTC‐binding factor (CTCF) and Brother of Regulator of Imprinted Sites (BORIS) motifs were enriched at breed‐specific boundaries, with motif frequency decreasing with distance from boundary centers, consistent with their roles in boundary formation and insulation (Figure S11L, Supporting Information).

Together, these findings indicate that compartment switching and TAD boundary remodeling contribute to distinct transcriptional programs associated with oxidative versus glycolytic fiber identity in lean‐type and Chinese indigenous pigs.

### 2.5 Promoter–Enhancer Interactions Orchestrate Breed‐Specific Transcriptional Programs Influencing Muscle Fiber Transformation

**FIGURE 5:**
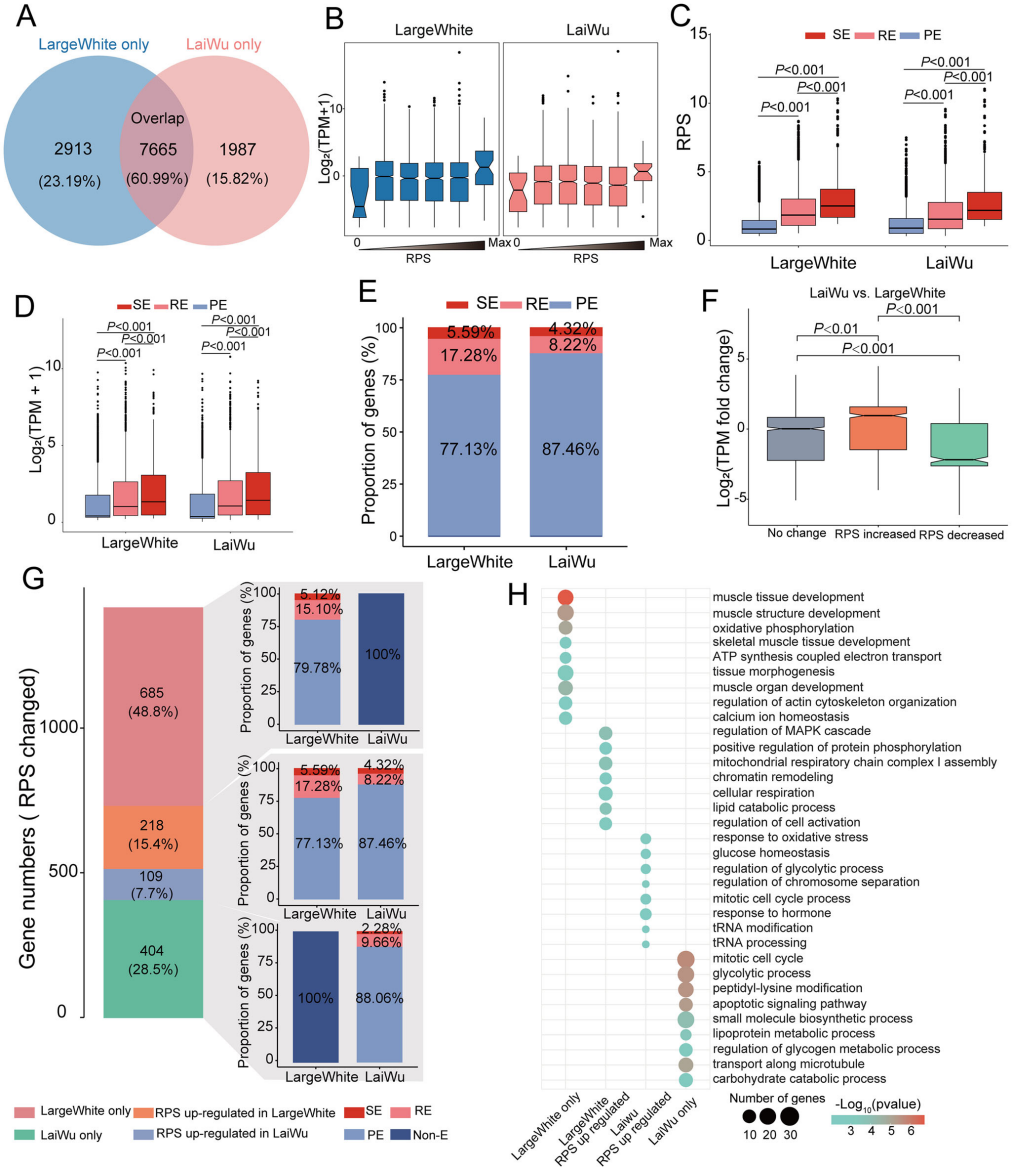
Rewiring of PEIs in Chinese and lean‐type pigs. (A) Overlap of genes associated with PEIs in LargeWhite and LaiWu pigs. (B) Genes with higher RPS exhibit increased expression levels. (C) RPS values of genes interacting with SEs, REs, and PEs. (D) Expression levels of genes interacting with SEs, REs, or PEs in LargeWhite and LaiWu pigs. (E) Proportion of PEI‐associated genes interacting with SEs, REs, and PEs in LargeWhite and LaiWu pigs. (F) Expression differences in genes with varying RPS values between LargeWhite and LaiWu pigs. (G) K‐means clustering was applied to genes with RPS changes in LargeWhite and LaiWu pigs (k = 4). H) Most enriched GO‐BP terms for genes with high RPS in each cluster. *p* values were calculated using the two‐sided Wilcoxon rank‐sum test in (C, D, F).

Enhancer–promoter interactions (PEIs) are fundamental to gene regulation and may contribute to muscle fiber‐type transformation [43, 44]. We generated a genome‐wide catalog of PEIs in skeletal muscle to assess their contribution to muscle fiber composition. Using PSYCHIC, we identified 29 355 high‐confidence PEIs (10 578 genes) in LargeWhite pigs and 27 368 (9652 genes) in LaiWu pigs (FDR ≤ 0.0001), with median interaction spans of ∼445 and ∼455 kb, respectively (Figure 5A, Figure S12A, Table S3, Supporting Information). Over 86% of PEIs were located within TADs and chromatin loops, and >64% involved long‐range promoter contacts, highlighting their spatial complexity (Figure S12B–E, Supporting Information).

To quantify PEI‐associated regulatory input, we calculated a regulatory potential score (RPS) for each gene. Genes with higher RPS values exhibited higher expression levels, consistent with additive contributions of multiple enhancers (Figure 5B). We next examined H3K27ac signal at PEI‐linked enhancers to stratify enhancer classes. Genes interacting with super‐enhancers (SEs; high H3K27ac) (∼4.96%) showed higher RPS and expression than genes linked to regular enhancers (REs; moderate H3K27ac) (∼12.75%) or poised enhancers (PEs; lacking H3K27ac) (∼82.29%) (Figure 5C–E).

We further analyzed transcription factor motif enrichment within PEI‐associated OCRs, revealing breed‐specific patterns (Figure S12F, Supporting Information). The PEIs of 2‐week‐old LargeWhite pigs were enriched for MEF2C motifs, consistent with oxidative fiber programs. Similarly, ATAC footprint analysis showed that MEF2C exhibited distinct binding patterns in LargeWhite pig‐specific PEIs, with higher binding scores in LargeWhite pigs than in LaiWu pigs (Figure S12G, Supporting Information). In contrast, Promoter‐Enhancer Interactions (PEIs) in 2‐week‐old LaiWu pigs were enriched for motifs from E‐twenty‐six (Ets) family members (e.g., E74‐like factor 1 (ELF1), E74‐like factor 2 (ELF2), and E74‐like factor 3 (ELF3)), PR domain zinc finger protein 1 (PRDM1), and THAP domain containing 11 (THAP11). Moreover, convergent CTCF–CTCF loops consistent with PEI orientation accounted for >56% of all detected loops (Figure S12H, Supporting Information), suggesting preferential localization of CTCF motifs near promoters and enhancers to facilitate PEI formation in both breeds (Figure S12I,J, Supporting Information).

Additionally, inter‐breed differences in regulatory potential scores (RPS) were associated with concordant changes in gene expression (Figure 5F). K‐means clustering identified four patterns among 1416 genes (|log_2_FC| > 1.5 and |ΔRPS| > 1), each characterized by distinct RPS profiles and corresponding changes in enhancer activity (Figure 5G). Functional enrichment analysis indicated that these clusters captured breed‐dependent regulatory programs consistent with the physiological divergence between lean‐type and Chinese indigenous pigs (Figure 5H). Notably, 685 genes exhibited LargeWhite–specific RPS patterns (e.g., *NDUFB4* (NADH:ubiquinone oxidoreductase subunit B4), *COA6* (cytochrome c oxidase assembly factor 6), and *COX4I1* (cytochrome c oxidase subunit 4I1)) [42] and were enriched for oxidative phosphorylation, ATP synthesis, muscle development, and calcium ion homeostasis, supporting the predominance of oxidative programs in 2‐week‐old LargeWhite pigs. By contrast, 327 genes displayed divergent RPS values between breeds. Of these, 218 showed higher RPSs in LargeWhite pigs (e.g., *NDUFAF2* (NADH:ubiquinone oxidoreductase complex assembly factor 2), *NDUFA2* (NADH:ubiquinone oxidoreductase subunit A2), and *NUBPL* (NUBP iron‐sulfur cluster assembly factor)) [42] and were enriched for mitochondrial respiratory chain complex I assembly, MAPK signaling, and kinase activity, whereas 109 exhibited higher RPSs in LaiWu pigs (e.g., *ZBTB20* (zinc finger and BTB domain containing 20) [45]) and were enriched for oxidative stress response, glycolysis, and glucose homeostasis. Additionally, 404 genes showed unique RPS values (e.g., *PPP1R3G* (protein phosphatase 1 regulatory subunit 3G), *PPP1R3F* (protein phosphatase 1 regulatory subunit 3F)) [46] in 2‐week‐old LaiWu pigs, linked to glycolytic processes, lipoprotein metabolism, and regulation of glycogen metabolic process, indicating the dominance of glycolytic muscle fibers in Chinese indigenous pigs (Figure 5H). qPCR further validated breed‐dependent expression of representative oxidative‐ and glycolysis‐related genes, supporting a link between PEI rewiring and transcriptional output (Figure S12K, Supporting Information). Collectively, these results indicate that breed‐specific PEI architecture contributes to transcriptional divergence underlying oxidative versus glycolytic muscle programs in lean‐type and Chinese indigenous pigs.

### 2.6 Super‐Enhancer‐Promoter Interaction Regulates PPP3CB by Modulating MEF2C Binding in Skeletal Muscle

**FIGURE 6:**
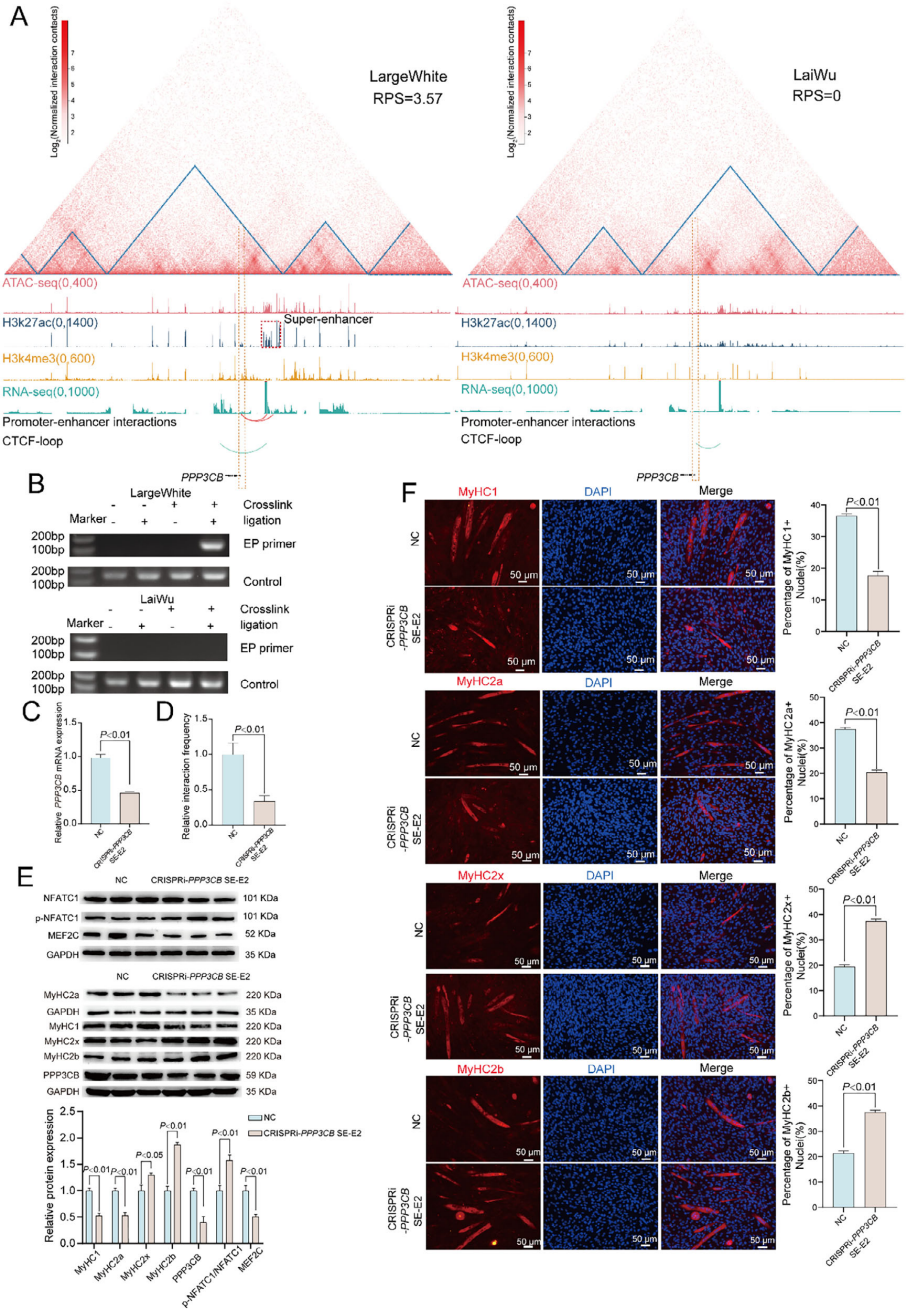
Super‐enhancer–promoter interaction regulates *PPP3CB* to promote oxidative fiber programs. (A) Multi‐omics tracks at the *PPP3CB* locus in LargeWhite and LaiWu pigs. (B) 3C assay validating interaction between the *PPP3CB* promoter and super‐enhancer (SE) E2. (C) qPCR analysis of *PPP3CB* mRNA after CRISPRi targeting *PPP3CB* SE‐E2 (n = 3 per group). (D) 3C‐qPCR analysis of interaction frequency after CRISPRi targeting *PPP3CB* SE‐E2 (n = 3 per group). (E) Changes in PPP3CB and indicated proteins after CRISPRi targeting *PPP3CB* SE‐E2; protein levels were normalized to GAPDH and expressed relative to the control group (n = 3 per group). (F) Left: Immunofluorescence staining for MyHC1, MyHC2a, MyHC2x, and MyHC2b in control and CRISPRi *PPP3CB* SE‐E2 groups. Right: Quantification of marker‐positive cells (n = 3 per group). Data are presented as mean ± SD from at least three independent experiments. *p* values were calculated using a two‐sided unpaired Student's *t*‐tests.

Comparative multi‐omics analyses revealed significantly stronger ATAC‐seq, H3K27ac, H3K4me3, and RNA‐seq signals in the vicinity of the *PPP3CB* transcription start site (TSS) in lean‐type pigs compared with Chinese indigenous pigs, consistent with higher *PPP3CB* transcriptional activity in lean‐type pigs (Figure S13A, Supporting Information). This observation was corroborated by Western blotting, which showed higher PPP3CB protein levels in lean‐type pigs (Figure S13B, Supporting Information). Moreover, Hi‐C analysis revealed an H3K27ac‐enriched super‐enhancer (SE) upstream of *PPP3CB* in LargeWhite pigs that physically contacts the promoter via chromatin looping, whereas this SE‐promoter interaction was not detected in LaiWu pigs (Figure 6A). Across four breeds, IGV visualization of epigenomic profiles further showed that this upstream region was annotated as an SE in both lean‐type breeds (LargeWhite and Duroc) but was absent in the Chinese indigenous breeds (JianLi and LaiWu) (Figure S15A, Supporting Information), supporting a lean‐type–associated regulatory element at the *PPP3CB* locus.

To further explore the promoter and super‐enhancer activity, we performed luciferase assays. The results demonstrated robust activity for the *PPP3CB* promoter (Figure S14A, Supporting Information). Furthermore, the assays identified the E2 segment as the core functional domain of the super‐enhancer (Figure S14B, Supporting Information). 3C (chromatin conformation capture) experiments confirmed a physical loop between the *PPP3CB* promoter and the super‐enhancer in lean‐type pigs, whereas this interaction was undetectable in Chinese indigenous pigs (Figure 6B, Figure S15B, Supporting Information). CRISPR interference (CRISPRi)‐mediated repression of the E2 segment significantly reduced *PPP3CB* mRNA abundance (Figure 6C), and 3C‐qPCR further revealed reduced promoter–super‐enhancer contact frequency (Figure 6D). Consistently, PPP3CB, MyHC1, MyHC2a, and MEF2C protein levels decreased, whereas MyHC2x, MyHC2b, and phosphorylated nuclear factor of activated T cells 1 (NFATC1) (p‐NFATC1) increased, with total NFATC1 unchanged (Figure 6E). Immunofluorescence staining showed that E2‐targeting CRISPRi reduced the proportion of MyHC1‐ and MyHC2a‐positive fibers and increased the proportion of MyHC2b‐ and MyHC2x‐positive fibers (Figure 6F). These changes were independently supported by quantification of fiber‐type marker–positive myotubes and Western blot analyses in Duroc (Figure S15C,D, Supporting Information). Similar trends were also observed upon *PPP3CB* knockdown in LargeWhite (Figure S16A,B, Supporting Information).

**FIGURE 7:**
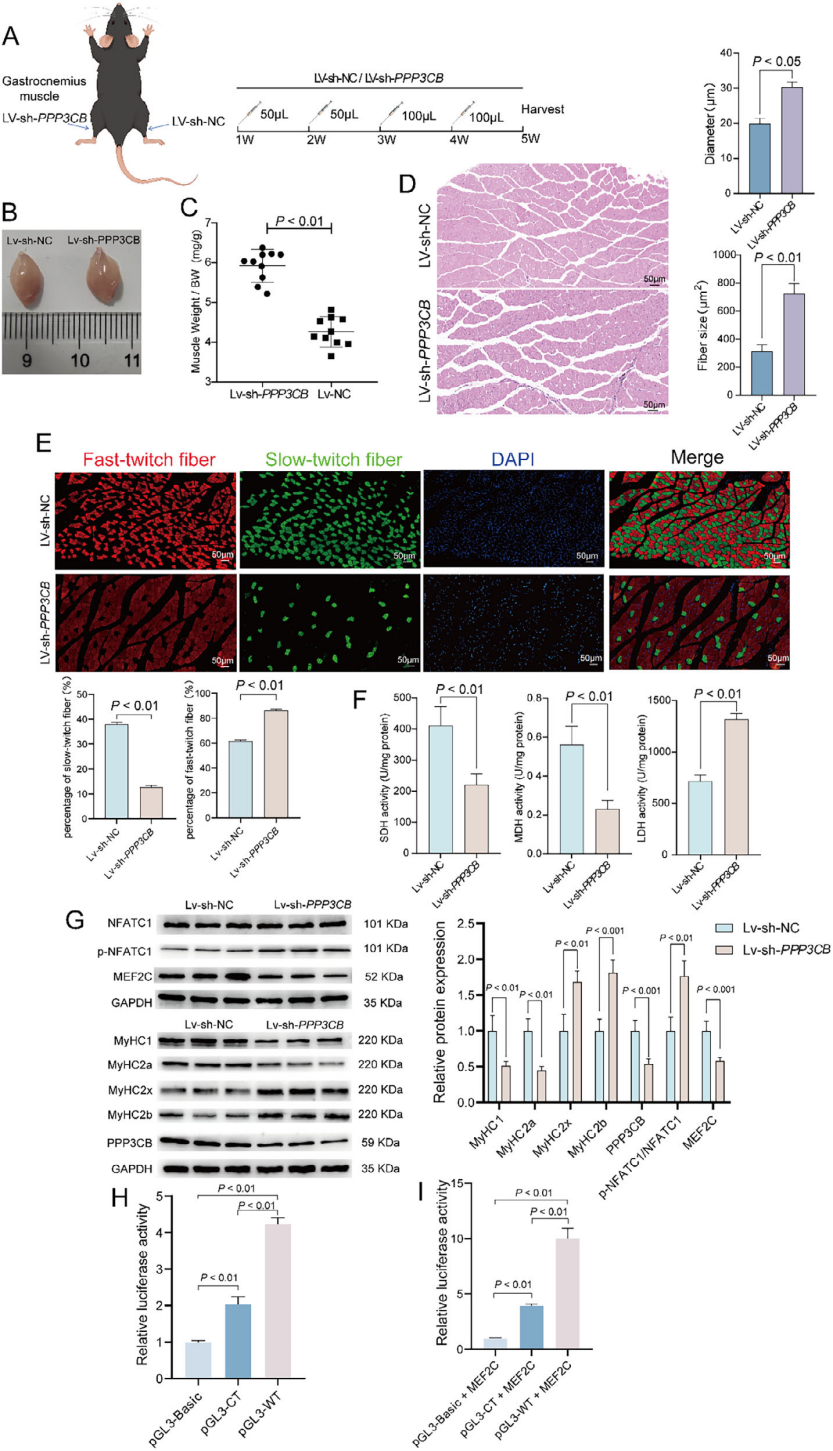
The effect of lentivirus‐mediated *PPP3CB* knockdown on muscle fiber types in mice at the in vivo level. (A) Lentivirus‐mediated *PPP3CB* knockdown, in vivo injection experiment schematic. (B) Representative gross morphology of gastrocnemius (Gas) muscles from the LV‐sh‐NC (control) and LV‐sh‐*PPP3CB* groups. (C) Quantification of ten independent experiments showed that *PPP3CB* knockdown via lentivirus significantly increased Gas muscle weights (n  =  10 per group). *p* values were calculated using a paired *t*‐test, and data were normalized to body weight (BW) (mg/g). (D) Representative H&E‐stained sections of Gas muscles from LV‐sh‐NC and LV‐sh‐*PPP3CB* mice (n = 3 per group). For morphometric analysis, ≥150 myofibers were quantified per mouse, and per‐mouse means were used for statistical analysis. Scale bar, 50 µm. (E) Representative immunofluorescence staining of fast‐ and slow‐twitch fibers in Gas muscles from 2‐month‐old mice injected with LV‐sh‐NC or LV‐sh‐*PPP3CB* (n = 3 per group). ≥150 myofibers were quantified per mouse, and per‐mouse means were used for statistical analysis. Scale bar, 50 µm. (F) Enzyme activities of SDH, MDH, and LDH in the Gas muscles from 2‐month‐old mice injected with LV‐sh‐NC or LV‐sh‐*PPP3CB* (n = 3 per group). (G) Western blot analysis and densitometric quantification of PPP3CB and indicated proteins in Gas muscles following lentivirus‐mediated *PPP3CB* knockdown (n = 3 per group). Band intensities were normalized to GAPDH and expressed relative to the control group. (H) Effect of base deletion on the promoter activity of Chinese indigenous and lean‐type pigs (n  =  3 per group). I) The base deletion is located at the MEF2C binding site, and overexpression of MEF2C affects promoter activity. WT represents lean‐type pigs *PPP3CB* promoter (712–1003 bp), and CT represents Chinese indigenous pigs *PPP3CB* promoter (712–1003 bp) (n  =  3 per group). Relative protein levels were normalized to GAPDH in the control group. Data are presented as mean ± SD. *p* values were calculated using a two‐sided Student's *t*‐test.

*PPP3CB* overexpression significantly increased the protein levels of PPP3CB, MyHC1, MyHC2a, and MEF2C, whereas MyHC2x, MyHC2b, and phosphorylated NFATC1 (p‐NFATC1) were reduced; total NFATC1 remained unchanged (Figure S16C, Supporting Information). Immunofluorescence staining corroborated an increase in MyHC1‐ and MyHC2a‐positive fibers and a concomitant decrease in MyHC2b‐ and MyHC2x‐positive fibers relative to controls (Figure S16D, Supporting Information). To assess functional conservation, we compared PPP3CB amino acid sequences across pig, mouse, human, bovine, and ovine species and found 99% similarity between pig and mouse (Figure S17A, Supporting Information). Given this high conservation, we investigated *PPP3CB* function in vivo by injecting LV‐sh‐NC or LV‐sh‐*PPP3CB* into the gastrocnemius of 4‐week‐old wild‐type (WT) mice (Figure 7A). *PPP3CB* knockdown increased gastrocnemius size and mass (Figure 7B,C) and increased myofiber diameter and cross‐sectional area, as assessed by H&E staining (Figure 7D). Immunofluorescence analysis indicated a shift toward a higher proportion of fast‐twitch fibers within the gastrocnemius (Figure 7E). Consistently, *PPP3CB* knockdown decreased oxidative enzyme activities (SDH and MDH) but increased glycolytic LDH activity (Figure 7F). Western blotting showed increased MyHC2b, MyHC2x, and p‐NFATC1 and decreased MyHC1, MyHC2a, and MEF2C (Figure 7G). Together, these results support a role for PPP3CB in promoting the transformation from glycolytic/intermediate toward oxidative fiber programs, potentially through the NFATC1–MEF2C axis.

Transcription factor binding motifs were analyzed using the JASPAR and AnimalTFDB 4.0 databases, identifying MEF2C binding motifs within the core functional domain E2 of the *PPP3CB* super‐enhancer and its promoter (Figure S14C, Supporting Information). Both qPCR and Western blot analyses confirmed that *MEF2C* overexpression increased *MEF2C* and *PPP3CB* expression, whereas siRNA‐mediated *MEF2C* knockdown decreased their expression (Figure S14D–F, Supporting Information). Dual‐luciferase reporter assays and CUT&Tag qPCR demonstrated that MEF2C bound to both the E2 domain and the *PPP3CB* promoter (Figure S14G–I, Supporting Information). Based on WGS and Sanger sequencing, we identified two *PPP3CB* promoter variants that distinguish Chinese indigenous and lean‐type pigs, comprising a SNP and an indel (Figure S17B, Supporting Information). Luciferase reporter assays showed that the SNP at position 793 did not significantly affect promoter activity between the Chinese indigenous and lean‐type alleles (Figure S17C, Supporting Information). By contrast, the deletion allele in Chinese indigenous pigs (CT) reduced promoter activity compared with the lean‐type allele (WT) (Figure 7H). Notably, this deletion overlaps a predicted MEF2C‐binding site, suggesting that sequence variation may modulate MEF2C‐dependent promoter activation (Figure 7I).

To assess the conserved function of MEF2C, we compared its amino acid sequences across pig, mouse, human, bovine, and ovine species, showing 98% similarity between pig and mouse MEF2C (Figure S17D, Supporting Information). MEF2C's effect on muscle fiber type transformation was further examined by injecting LV‐sh‐NC and LV‐sh‐*MEF2C* into the gastrocnemius muscles of 4‐week‐old wild type mice (Figure S18A, Supporting Information). *MEF2C* downregulation significantly increased gastrocnemius size and weight (Figure S18B,C, Supporting Information), and H&E staining revealed a marked increase in myofiber diameter and cross‐sectional area (Figure S18D, Supporting Information). Immunofluorescence staining revealed an increase in fast‐twitch fibers after *MEF2C* downregulation (Figure S18E, Supporting Information). Downregulation of *MEF2C* also reduced SDH and MDH activities and increased LDH activity (Figure S18F, Supporting Information). Western blot analysis confirmed increased levels of MyHC2b and MyHC2x, with a decrease in MyHC1 and MyHC2a fibers (Figure S18G, Supporting Information). These results suggest that MEF2C promoted the conversion of glycolytic and intermediate to oxidative muscle fibers through a positive feedback loop with *PPP3CB*.

## 3 Discussion

Muscle fiber composition is a defining trait in livestock production and muscle physiology; however, the regulatory mechanisms underlying its establishment during early postnatal development remain incompletely understood [11]. Previous studies have shown that epigenomic profiles from bulk tissue can closely recapitulate key features observed in purified muscle cell nuclei [20]. Building on this, we performed integrative multi‐omics profiling (ATAC‐seq, ChIP‐seq, RNA‐seq, WGS, and Hi‐C) of the LD muscle from 2‐week‐old lean‐type and Chinese indigenous pigs. This work provides an integrated multi‐omics view of breed‐associated variation in muscle fiber composition, revealing a multilayer regulatory architecture involving chromatin accessibility, histone modifications, 3D genome organization, and transcription factor occupancy. Our phenotypic and molecular analyses indicated that 2‐week‐old lean‐type pigs contained more oxidative fibers, whereas Chinese indigenous pigs contained more glycolytic fibers at the same stage. These observations are consistent with prior studies suggesting that genetic background shapes fiber‐type composition, with downstream consequences for meat quality and metabolic efficiency [47, 48, 49]. Importantly, the breed‐dependent divergence in enzyme activities and gene expression during early postnatal development suggests early‐life epigenetic programming of muscle fiber composition, although the extent to which later environmental exposures modulate these programs warrants further investigation.

Chromatin accessibility and histone modifications further defined breed‐specific regulatory landscapes. In particular, the markedly higher number of active enhancers and super‐enhancers in lean‐type pigs—especially at loci involved in oxidative metabolism and myofiber‐type specification—suggests a more transcriptionally permissive chromatin environment. For instance, previous studies have reported that perturbation of glycolysis‐associated Myc super‐enhancers is accompanied by c‐Myc upregulation and induction of PPARG coactivator 1 alpha (*PPARGC1A*) and peroxisome proliferator‐activated receptor‐delta (*PPAR‐δ*) expression, thereby enhancing oxidative metabolism [50]. These observations support a model in which oxidative fiber specification is reinforced by widespread enhancer activation and increased chromatin accessibility, potentially enabling robust transcription and sustained metabolic gene networks [51, 52]. By contrast, Chinese indigenous pigs exhibited regulatory signatures enriched for biosynthetic and glycolytic pathways, suggesting a distinct developmental programming trajectory.

RNA‐seq data showed a precise alignment between transcriptional output and epigenetic marks. Genes associated with oxidative phenotypes, such as *PPARGC1A* and *CAMK2A*, were not only highly expressed but also marked by high ATAC‐seq and H3K27ac signals. The combinatorial epigenomic model we propose demonstrates that transcriptional regulation is most effectively orchestrated by integrating multiple chromatin features, rather than relying on isolated marks. This aligns with previous findings in developmental systems where histone modifications and accessibility act synergistically to define cell fate [13, 15, 53]. The dynamic regulation of regulatory elements was further evidenced by breed‐specific distributions of enhancers and promoters, as well as motif composition. Super‐enhancers linked to key oxidative genes were particularly abundant in lean‐type pigs, with MEF2C, MEF2D, and NR5A2 motifs prevalent in the enhancer region. MEF2C, a well‐established regulator of oxidative muscle fiber development [54], was not only enriched at lean‐type specific enhancers but also involved in direct promoter‐enhancer loops, reinforcing its pivotal role in regulatory coordination. The contrast with the SOX family motifs, which are enriched in Chinese indigenous pigs, underscores the divergent transcriptional hierarchies that govern glycolytic muscle development [55, 56]. Furthermore, both pig breeds displayed a higher prevalence of breed‐specific SNPs and indels in enhancers specific to each breed compared to breed‐specific promoters. These findings highlight the crucial role of enhancers in breed‐specific traits and propose potential directions for future genetic research.

Hi‐C‐based analysis of chromatin compartments revealed that approximately 6% of the genome underwent A/B compartment switching, which significantly correlated with transcriptional changes. This suggests that compartment‐level chromatin remodeling is functionally relevant to fiber‐type‐specific gene expression. Genes that moved from inactive (B) to active (A) compartments, such as *CAMK1D* [39], were enriched in pathways supporting oxidative capacity, while the inverse was true for genes like *GPD1* involved in glycolysis [40]. These results corroborate studies in other tissues, which have shown that compartment transitions can reshape transcriptional profiles in response to developmental cues [15, 16, 17]. TAD boundary dynamics further highlighted the plasticity of 3D genome organization in defining breed‐specific gene regulation. Specifically, changes in TAD boundaries, especially boundary separation and fusion, influenced the expression of genes such as *FST*, which is known to regulate muscle fiber transformation [41]. These boundary shifts were associated with CTCF and BORIS motifs, consistent with their established roles in chromatin loop formation and insulation. Such alterations in genome topology likely underlie the differential accessibility and the probability of enhancer‐promoter contact observed across breeds. Moreover, promoter‐enhancer interactions (PEIs) were globally reprogrammed between breeds [57]. Genes with higher RPS levels showed increased expression, particularly those interacting with super‐enhancers. These genes were enriched in oxidative metabolic processes and muscle fiber type transformation, thus providing a mechanistic explanation for the elevated oxidative capacity observed histologically and transcriptionally. The differential wiring of PEIs underscores how regulatory topologies are fine‐tuned to breed‐specific myofiber type transformation programs.

Calcineurin plays a crucial role in the calcium‐dependent signaling pathway, particularly in skeletal muscle, where it regulates muscle fiber development, differentiation, hypertrophy, and the activation of the slow‐oxidative muscle fiber phenotype [58]. *PPP3CB*, the catalytic subunit of calcineurin, is a Ca^2^^+^/calmodulin‐dependent serine/threonine phosphatase [59]. The identification and functional validation of a super‐enhancer upstream of *PPP3CB* provide essential mechanistic insights. The super‐enhancer–*PPP3CB–*MEF2C feedback loop illustrates how epigenomic structure and transcription factor binding converge to control oxidative fiber identity. Knockdown and overexpression experiments, both in vitro and in vivo, validated the regulatory efficacy of this circuit. Additionally, a natural mutation in the MEF2C binding site within the *PPP3CB* promoter in Chinese pigs further links genetic variation to altered regulatory output. Aligning with previously reported works [57, 60], these findings highlight the evolutionary divergence of regulatory elements that directly modulate muscle physiology.

The MyHC isoform transformation from early life to adulthood suggests a breed‐dependent postnatal trajectories of muscle remodeling. This is suggested by comparing the patterns in MyHC isoform expression/protein abundance and related enzyme activities (SDH, MDH, and LDH) at two developmental time points (2 weeks and 180 days), which may contribute to divergent adult phenotypes. Longitudinal studies including additional intermediate stages and incorporating fiber‐level classification and quantification will be valuable to test this possibility and to identify potential drivers (e.g., activity, endocrine cues, and metabolic programming).

In summary, this study demonstrates that early muscle fiber differences in pigs arise from a multilayered epigenetic framework shaped by breed‐specific regulatory elements, chromatin architecture, and transcription factor circuits. This enhances our understanding of the genetic and epigenomic pathways governing myofiber type transformation and provides a theoretical basis for improving meat quality attributes.

## 4 Conclusion

**FIGURE 8:**
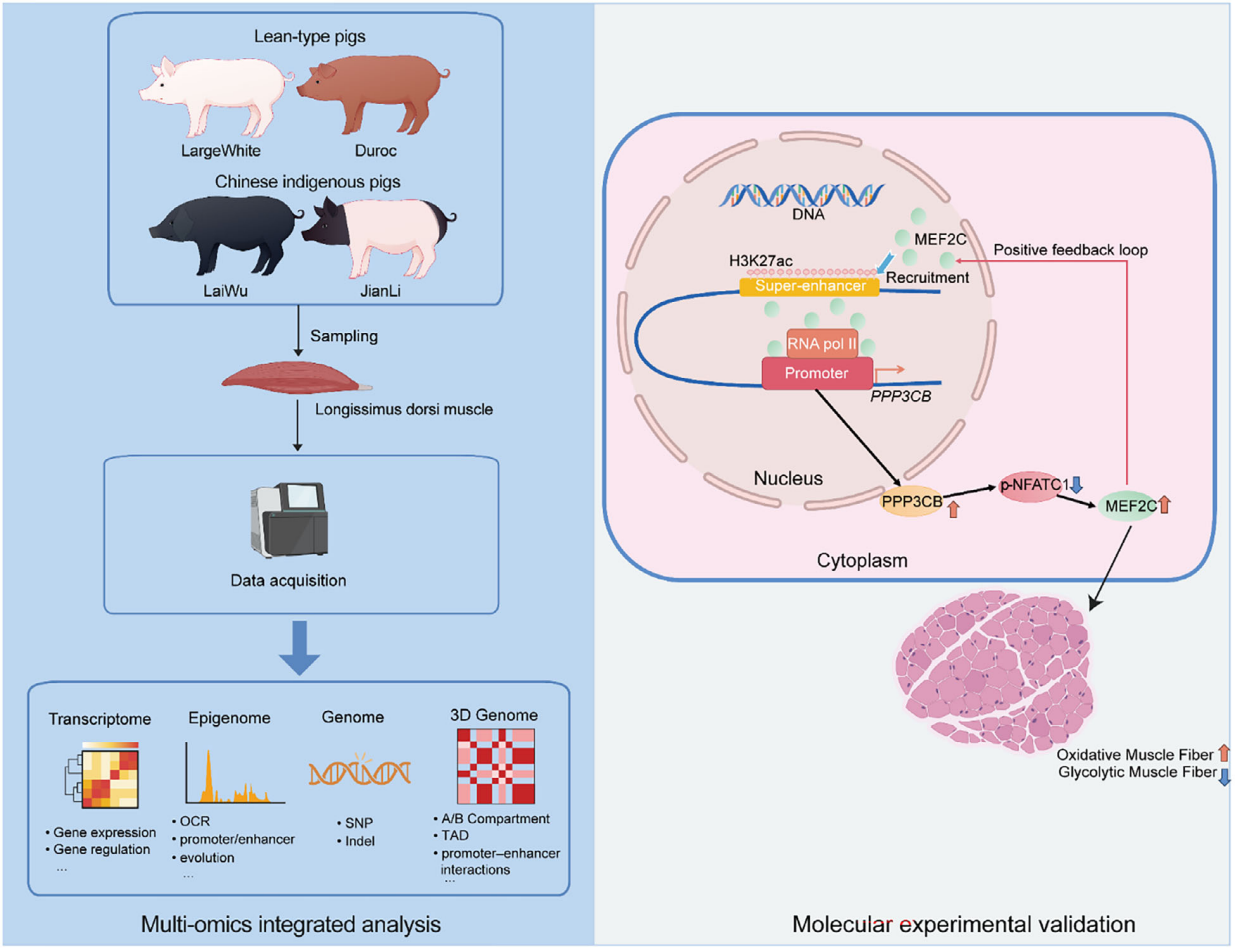
Schematic diagram depicting multi‐omics and molecular experimental validation analysis of muscle fiber type differences in lean‐type and Chinese indigenous pigs. This study uses a multi‐omics approach to investigate the regulatory mechanisms underlying differences in muscle fiber composition between lean‐type and Chinese indigenous pigs. Through RNA‐seq, ATAC‐seq, ChIP‐seq, WGS, and Hi‐C, we identified distinct epigenomic landscapes, genetic variation maps, and 3D genome structures associated with oxidative and glycolytic fiber programs. Key findings include breed‐specific super‐enhancers, compartment transitions, and promoter–enhancer rewiring. Notably, a lean‐specific super‐enhancer upstream of *PPP3CB* interacts with MEF2C to promote oxidative fiber identity. Created with BioRender.com.

This study provides an integrative multi‐omics framework that uncovers the regulatory basis of early muscle fiber‐type divergence between lean‐type and Chinese indigenous pigs. By combining RNA‐seq, ATAC‐seq, ChIP‐seq, WGS, and Hi‐C analyses, we identified breed‐specific deployment of cis‐regulatory elements, differential compartment transitions, dynamic TAD boundaries, and promoter–enhancer interactions that regulate gene expression variability and affect muscle fiber composition. Importantly, we uncovered a regulatory circuit involving a lean‐specific super‐enhancer upstream of *PPP3CB*, which interacts with MEF2C to promote oxidative fiber identity (Figure 8). These findings reveal the chromatin and spatial genomic logic underpinning muscle phenotypic diversity and provide a molecular foundation for the targeted improvement of meat quality traits through genomic and epigenomic strategies.

## 5 Experimental Section

### 5.1 Animals, Ethical Approval, and Sample Collection

All pigs were maintained under strictly standardized husbandry conditions to minimize non‐genetic variability. Specifically, 2‐week‐old (postnatal day 14) male piglets from four breeds (two Chinese Indigenous breeds: JianLi and LaiWu; and two lean‐type breeds: Duroc and LargeWhite) and 180‐day‐old adult pigs from two breeds (LargeWhite and JianLi) were acquired from Huazhong Agricultural University's Experimental Pig Farm. For the 14‐day‐old piglets and 180‐day‐old adult pigs, all animals were housed in the same climate‐controlled farrowing house, with consistent temperature and humidity maintained throughout the rearing period. Adult pigs were given free access to feed and water, while piglets were exclusively breastfed, ensuring uniform early nutrition. C57BL/6 male mice were obtained from the Institutional Laboratory Animal Center of Huazhong Agricultural University (HZAU), where all experimental procedures were carried out. The mice were provided with ad libitum access to feed and water throughout the study.

LD muscle samples were collected from 2‐week‐old male pigs of each breed and from the 180‐day‐old adult LargeWhite and JianLi pigs. Each sample was processed according to a standard protocol. One portion was fixed in 4% paraformaldehyde for histological analysis. Another portion was snap‐frozen in liquid nitrogen for subsequent molecular analyses. The remaining tissue was stored at −80°C for RNA and protein extraction and for enzyme activity assays. All animal experiments were approved by the Institutional Animal Care and Use Committee (IACUC) of HZAU (Permit Number: HZAUSW‐2017‐008).

### 5.2 Clarification of Cohorts

The animals described above were used for phenotypic measurements and validation assays in this study. The omics sequencing datasets used for integrative analyses were generated previously by our group and collaborators as part of earlier projects and have been deposited in public repositories; accession IDs and detailed metadata are provided in Table S1. These omics datasets were generated from 2‐week‐old piglets raised at the same Experimental Pig Farm under the same standardized pre‐weaning husbandry conditions described above.

### 5.3 Phenotypic Measurement

The samples fixed in 4% paraformaldehyde were washed with running water, dehydrated using an ethanol series, cleared in xylene, and embedded in paraffin wax. The specimens were sectioned to a thickness of 4 µm using a Leica RM2235 microtome (Leica Instrument Company). Muscle sections were stained using established techniques with hematoxylin and eosin (H&E), and histological images were visualized under a light microscope [25]. For immunofluorescence staining of muscle sections, the samples were treated with 0.01 M sodium citrate (pH 6.0) at 70°C, then permeabilized with 0.1% Triton X‐100. Sections were incubated overnight at 4°C with the primary antibodies anti‐dystrophin (Abcam, UK, ab275391, 1:200), anti‐slow myosin skeletal heavy chain (Sigma, USA, M8421, 1:1000), and anti‐fast myosin skeletal heavy chain (Servicebio, China, GB112130, 1:1000) after blocking at 37°C with a buffer (P0100, Beyotime, China). For quantitative histological analysis, H&E‐stained sections were used for morphological evaluation, and muscle fiber diameter was measured using ImageJ based on the minimal Feret's (equivalent) diameter. To ensure accurate boundaries, dystrophin immunofluorescence staining was performed to outline the sarcolemma and define muscle fiber borders. ImageJ was then used to measure the cross‐sectional area (CSA) of each fiber based on these boundaries. The composition of fast and slow muscle fibers was determined by immunofluorescence for slow and fast myosin heavy chain (MyHC), with ImageJ used to analyze the area of fast (red) and slow (green) fibers, calculating their proportions. For each biological replicate (one animal), at least three independent muscle sections were analyzed with multiple non‐overlapping fields quantified, ensuring a minimum of 150 fibers per replicate. The mean of each biological replicate was used for statistical analysis.

Subsequently, the secondary antibodies (Proteintech, China, anti‐mouse CY3, anti‐rabbit CY3, anti‐rabbit FITC) were used after washing with PBS. A fluorescence microscope (IX51‐A21PH; Olympus, Tokyo, Japan) was used to acquire images. Commercial kits (Jiancheng, China) for measuring lactate dehydrogenase (LDH), succinate dehydrogenase (SDH), and malate dehydrogenase (MDH) activities in muscle tissue were used according to the manufacturers' instructions (LDH: A020‐2‐2, SDH: A022‐1‐1, MDH: A021‐2‐2).

### 5.4 Mixed‐Effects Model Analysis

To assess multiple phenotypic and biochemical readouts while accounting for inter‐individual variation, we applied a linear mixed‐effects model using the lme4 package in R [61]. The model was specified as:

$$\begin{array}{ccc} \mathrm{Value}\_\mathrm{scaled} & ∼ & \mathrm{breed}\;\mathrm{type}\times \mathrm{Indicator}+\mathrm{BodyWeight}\\ & & +\left(1|\mathrm{Individual}\right) \end{array}$$

In this model, breed type contrasts lean‐type versus Chinese indigenous pigs, BodyWeight is included as a covariate, and Individual accounts for repeated measurements within the same biological replicate. Marginal means were estimated using emmeans [62], and pairwise comparisons were performed with multiple comparison corrections. For this analysis, the relative grayscale values of MyHC1, MyHC2a, MyHC2x, and MyHC2b were derived from Western blot analysis and normalized to GAPDH to quantify protein abundance for each Myosin Heavy Chain isoform. MDH, SDH, and LDH enzyme activities. Slow‐twitch % is calculated by determining the proportion of the green fluorescence area of MyHC1‐positive fibers in muscle cross‐sections relative to the total fiber area (red and green fluorescence).

### 5.5 Total RNA Extraction, Reverse Transcription, and Quantitative Real‐Time PCR (qRT‐PCR)

Total RNA was extracted using TRIzol reagent (Invitrogen, USA). RNA concentration and quality were determined using a NanoDrop 2000 (Thermo, USA) and agarose gel electrophoresis, respectively. 1 µg of total RNA was reverse transcribed using the PrimeScript RT reagent kit with gDNA Eraser (Takara, Japan). qRT‐PCR analysis was performed using a LightCycler 480 II (Roche, Switzerland) system. The primers used are listed in Table S4. Relative RNA expression levels were calculated using the Ct (2–^ΔΔCt^) method.

### 5.6 Western Blotting

Tissues and cells were lysed in RIPA buffer containing protease and phosphatase inhibitors, following the manufacturer's protocol (P0013B, Beyotime, China). The protein samples were then heated at 95°C for 10 min in a 5× sodium dodecyl sulfate (SDS) sample buffer, and the proteins were separated via SDS‐PAGE (30 µg per lane). After electrophoresis, the proteins were transferred to polyvinylidene difluoride (PVDF) membranes (Millipore, IPVH00010, USA) using a Mini Trans‐Blot Cell system (Bio‐Rad, USA). The membranes were blocked with 5% non‐fat milk for 2 h at room temperature. Subsequently, the membranes were incubated overnight at 4°C with primary antibodies against MyHC2a (Santa Cruz, USA; sc‐53095; 1:1000), MyHC2b (Invitrogen, USA, 14‐6503‐80, 1:1000), MyHC1 (Santa Cruz, USA; sc‐53090; 1:500), MyHC2x (DSHB, USA; 6H1; 1:500), PPP3CB (Proteintech, China; 13340‐1‐AP; 1:5000), NFATC1 (Proteintech, China; 66963‐1‐Ig; 1:2000), Phospho‐NFATC1 (Zenbio, China; R25143; 1:2000), MEF2C (Abcam, USA; ab211493; 1:1000), and GAPDH (Proteintech, China; 10494‐1‐AP; 1:5000). The membrane was then incubated with HRP‐conjugated secondary antibodies (Servicebio, China; GB23301 or GB23303; 1:3000) for 1 h at room temperature. Protein bands were visualized using ECL (Bio‐Rad, USA).

### 5.7 RNA‐seq Library Preparation, Sequencing, and Analyses

LD muscles were collected from 2‐week‐old JianLi and LaiWu male pigs, with 2 biological replicates per breed. Total RNA was extracted using TRIzol reagent (Thermo Scientific, 15596026). Strand‐specific RNA‐seq libraries were prepared using the Illumina method and sequenced on the Illumina HiSeq X Ten system (PE150). Sequencing reads generated in this study were analyzed together with publicly available RNA‐seq datasets from the LD muscle of 2‐week‐old LargeWhite and Duroc pigs. All reads were then processed with Trim_galore (v0.6.10) to remove low‐quality sequences. High‐quality sequencing data were matched to the pig reference genome (Sscrofa11.1) utilizing STAR (v2.6.0c) [63]. Only uniquely mapped reads were analyzed and quantified using RSEM (v1.3.3), with gene expression assessed based on TPM (Transcripts Per Million) values for each transcript [64]. A Spearman correlation study was conducted across the various breeds. Differentially expressed genes (DEGs) were identified with DESeq2 [65] based on read count data, with Padj < 0.05 and |log_2_foldchange| > 1.5.

### 5.8 ChIP‐seq Library Preparation, Sequencing, and Analyses

The LD muscles were taken from JL and LW pigs at the age of 2 weeks, and two biological replicates were taken from each breed. The frozen tissue samples were ground into a fine powder using liquid nitrogen. Chromatin was crosslinked at room temperature for 20 min using 1% formaldehyde (Sigma‐Aldrich, 252549). 0.2 M Glycine was added to quench the crosslinking reaction. Genomic DNA was sheared using Covaris S220. Immunoprecipitation was performed by incubating M‐280 sheep anti‐rabbit IgG Dynabeads (ThermoFisher, 11203D) with 3 µg of antibodies targeting H3K4me3 (Millipore, 04–745) or H3K27ac (Abcam, ab4729) at 4°C for 4 h. The chromatin (30 µg) was incubated overnight at 4°C with the antibody‐bead complexes to enrich the targeted histone modifications. ChIP‐seq libraries were prepared following slight modifications to the Illumina protocol and sequenced on the Illumina HiSeq X Ten platform using paired‐end sequencing (PE150). ChIP‐seq obtained the data and co‐analyzed with the publicly available ChIP‐seq data of the LD muscle of two 2‐week‐old (LargeWhite and Duroc). All raw reads were processed using Trim_galore (v0.6.10) to remove low‐quality segments. A pig reference genome (Sscrofa11.1) was used to align the remaining high‐quality reads with Bowtie2 (v2.2.6) using default settings. PCR duplicates were filtered using Samtools (v1.3.1). H3K27ac and H3K4me3 peaks were identified using MACS2 [66]. Peak annotation was performed using ChIPseeker with the default parameters [67]. The ROSE algorithm was used to identify super‐enhancers (SEs) [68]. Areas outside 12.5 kb that were identified as SEs by combining H3K27ac ChIP‐seq peaks were then prioritized based on H3K27ac signal intensity. Super‐enhancers (SEs) are defined as peaks above the inflection point, whereas regular enhancers (REs) are defined as peaks below it. The poised enhancers region (PE) interacts with a distal promoter but does not coincide with an H3K27ac peak.

### 5.9 ATAC‐seq Library Preparation, Sequencing, and Analyses

Approximately 5 mg of LD muscle tissue from 2‐week‐old JianLi and LaiWu male pigs was homogenized into a fine powder using liquid nitrogen, with two biological replicates per breed. The powdered tissue was reconstituted in 1 mL of cold PBS and centrifuged to separate components. Cell pellets were resuspended in a lysis buffer containing HEPES, NaCl, EDTA, glycerol, NP‐40, and Triton X‐100. Nuclei were isolated using a modified method, yielding ∼50 000 nuclei per sample. A transposition reaction mixture (TD buffer, ddH_2_O, TDE) was added to the nuclei and incubated at 37°C for 1 h. DNA was purified using the QIAGEN MinElute PCR Kit. Transposed fragments were amplified via PCR, and fragments ranging in size from 100 to 600 bp were selected by gel purification. The purified products were sequenced on the Illumina HiSeq X Ten platform (PE150). The data obtained by ATAC‐seq were co‐analyzed with publicly sourced ATAC‐seq data from the LD muscle of 2‐week‐old LargeWhite and Duroc male pigs. Raw sequencing data were subjected to quality control using Trim_galore (v0.6.10). After trimming, Bowtie2 (v2.2.6) was used with default parameters to align the reads to the pig reference genome (Sscrofa11.1). RemoveChrom was used to eliminate mitochondrial alignments, and Picard (v1.126) was used to remove duplicate reads. Using MACS2 and the parameters “‐nomodel ‐extsize 200 ‐shift ‐100,” a set of prominent peaks was identified. The association between these peaks and A/B compartments was investigated, as was the enrichment of ATAC peaks next to transcription start sites. To identify breed‐specific peaks, we combined all data peaks into a consensus set. By subtracting the background noise from the ATAC signal strength (normalized read count per base), enrichment levels were ascertained. The DESeq2 R tool was used to identify breed‐specific peaks, and significant peaks were defined as |log_2_foldchange|>1 and FDR<0.05. HOMER's findMotifsGenome.pl (v4.10.3) [69] using default parameters, retaining motifs with a *p* value below 0.01. The vertebrate TF motifs were downloaded from JASPAR (2024) [70]. Binding scores for each TF and differential TF binding analysis were conducted using TOBIAS with default parameters [71]. Only TFs with a TPM of 0.5 or higher in at least one sample were considered expressed and included in subsequent analysis.

### 5.10 Whole Genome Sequencing Analysis

Whole‐genome sequencing (WGS) raw reads of four pig breeds (six biological replicates each) were retrieved from public repositories. All reads were initially filtered using Trim_galore (v0.6.10) to eliminate low‐quality sequences. Paired‐end reads obtained were then mapped to Sscrofa11.1 reference genome using BWA (v0.7.12) [72] with default parameters. The results were then sorted, and SAMtools (v1.9) [73] was used to create binary BAM files. SNP and Indel calling was done through the GATK (v4.1.4.1) [74] with the HaplotypeCaller module and joint genotyping in the GenotypeGVCFs module. Complex filtering was applied to ensure variant quality based on the following criteria: QUAL < 30.0, QD < 2.0, FS > 60.0, MQ < 40.0, SOR > 3.0, MQ RankSum < −12.5, and ReadPos RankSum < −20.0. SNP complex filtering used QD and FS, while Indel hard filtering used them as well. The VariantFiltration method of GATK was employed to perform all filtering steps.

### 5.11 Identification of Regulatory Elements and Phylogenetic Tree Construction

The overlap of H3K27ac and H3K4me3 consensus peaks was used to define regulatory regions (promoters and enhancers) using the intersectBed tool in bedtools v2.30.0. Promoters were defined by at least 50% overlap of H3K4me3 and H3K27ac, while H3K27ac marked enhancers without overlap with H3K4me3. Active regulatory elements (REs) specific to lean‐type pigs (LSP/LSE) and Chinese indigenous pigs (CSP/CSE) were identified, along with active common promoters (ACP) and enhancers (ACE). Breed‐specific SNPs and indels were also identified (LSS/LSI for lean‐type pigs, CSS/CSI for Chinese indigenous pigs). Gene expression levels and marker intensities (H3K4me3, H3K27ac, chromosomal accessibility) were analyzed in these regions. Pairwise distance matrices were calculated using Spearman's correlation coefficient (1−ρ). WGS data were integrated by extracting SNPs and indels, and additional distance matrices were generated through multiple sequence alignment (MSA). A phylogenetic tree was constructed using the neighbor‐joining method in the ape R library, with 1000 bootstrap replicates to assess branch‐length reliability [75].

### 5.12 In Situ Hi‐C Library Preparation, Sequencing and Analyses

The LD muscle tissue of a 2‐week‐old LaiWu male pig was cryogenically ground in liquid nitrogen, crosslinked in 2% formaldehyde for 10 min, and quenched with 0.2 M glycine for 5 min. It was resuspended in ice‐cold Hi‐C lysis solution (0.2% Igepal CA630, 10 mM Tris‐HCl, 10 mM NaCl) and incubated on ice for 20 min. After centrifugation, the pellet was resuspended in 50 µL of 0.5% SDS and heated at 62°C for 5–10 min for chromatin lysis. The mixture was neutralized with water and Triton X‐100, then incubated at 37°C. Chromatin digestion with MboI enzyme was done overnight at 37°C, followed by biotinylation of the ends using biotin‐14‐dATP, dCTP, dGTP, dTTP, and DNA Polymerase I. T4 DNA ligase was used to ligate the biotinylated fragments, and the mixture was incubated for 4 h. The ligated chromatin was fragmented with Covaris S220 to 300–500 bp. Sequencing libraries were prepared and sequenced on the Illumina HiSeq X Ten system (PE150). The Hi‐C sequencing data were co‐analyzed with publicly available Hi‐C data from the LD muscle of 2‐week‐old LargeWhite male pigs. The Hi‐C datasets were first filtered with Trim Galore (v0.6.10) to remove low‐quality sequences, and then analyzed with Juicer (v1.5) [76]. Initially, high‐quality Hi‐C reads were aligned to the pig reference genome (Sscrofa11.1) using Bowtie2 (v 2.2.6) [77]. The aligned read pairs were assigned to specific restriction motif fragments for further analysis. After removing duplicates, low‐quality pairs (MAPQ < 30) and intrafragment pairs, the remaining valid Hi‐C read pairs were retained for downstream analysis. Contact matrices were generated at a 100 kb resolution and underwent normalization using both the Knight‐Ruiz (KR) normalization technique [78] and the quantile normalization method [79]. The reliability and consistency of the Hi‐C data were assessed using the hicrep tool [80]. To estimate the 3D structure of the LargeWhite and LaiWu pig genomes, the PASTIS software (v0.3.3) was employed, utilizing a 25‐kb resolution matrix along with the multidimensional scaling (MDS) module for structural analysis [81].

### 5.13 A/B Compartments Identification

Compartment A and B were identified using the dcHiC tool [82], which extracted the first two principal components (PCs) from contact maps at 100 kb resolution. The relevant PC was chosen for further analysis, followed by normalization, sign correction, and comparison across different breeds. Transcriptional start sites, including all gene isoforms for each gene ID, were retrieved from the RefSeq database [83] to associate genes with specific compartments. Promoters were defined as regions 2 kb upstream and 500 bp downstream of the transcriptional start site. Bedtools intersect was used to calculate the overlap between these promoter regions and the A/B compartment bins. If promoters overlapped multiple bins, the bin with the most significant overlap was selected, and its corresponding compartment label was assigned to the gene. Visualizations of the contact maps were created using the WaSHU Epigenome Browser [84] and Juicebox [85].

### 5.14 Identification of Topologically Associated Domains

TADs were identified using HiCExplorer (v3.4.1) [86], which computes the distance between the left and right regions within each Hi‐C matrix bin using the TAD‐separation score. This score is derived from the average z‐score of interactions between the two regions (diamond‐shaped) within a sliding window of different dimensions. TADs are delineated as areas where the TAD‐separation score attains a local minimum. KR‐normalized matrices were utilized as input with default configurations, except for the addition of the ‘–correctForMultipleTesting fdr’ argument. To categorize a TAD's A/B status, we assessed the ratio of A/B bin assignments (PC1) within the TAD. TAD was classified as an A‐TAD if over 70% of its specified bins were located in the A compartment; otherwise, it was categorized as a B‐TAD. A distinct border is delineated as one recognized in a single breed. Genes situated at these borders, including those within or next to them, were observed to be affected by alterations in TAD placement.

### 5.15 Identification of Promoter‐Enhancer Interaction

Promoter‐enhancer interactions for each breed were found using methodologies described in prior studies [17, 18, 87]. To identify PEIs for each gene, Hi‐C readings from biological replicates at each step were aggregated, and contact matrices at a 25 kb resolution were constructed. The matrices were partitioned into smaller portions (20 Mb × 20 Mb) with a 10 Mb overlap and examined using the PSYCHIC program with default parameters to discover interactions significantly enriched in promoter regions [88]. PEIs were maintained if their false discovery rate (FDR) was below 0.01 and the interaction distance was 15 kb or more. To evaluate the influence of enhancers on gene regulation, the regulatory potential score (RPS) was computed for each gene, reflecting the aggregate of significant interaction intensities (Log_10_(observed contacts − anticipated contacts)) [17]. Differential RPS values were determined using the criteria |log_2_FC| > 1.5 and |ΔRPS| > 1.

### 5.16 Identification of CTCF‐CTCF Loops

FIMO (v5.1.1) [89] was employed to discover CTCF motif sites and their orientations in the pig reference genome, referencing the consensus CTCF motif from the JASPAR CORE 2024 vertebrate database [70]. The motifs were primarily located at TAD borders and enhancer areas, as anticipated. We focused on the LargeWhite and LaiWu breeds for loop identification, using HICCUPS at a 5 kb resolution with a q‐value cutoff of <0.05 [76]. Subsequently, a collaborative analysis was performed, integrating the genomic CTCF site data with the identified loops. The loops associated with CTCF were categorized into four groups based on their orientations.

### 5.17 Analysis of Functional Enrichment

Clusterprofiler [90] was used with default parameters to conduct enrichment analyses. Before submitting the gene lists for enrichment analysis, g: Profiler [91]was used to align pig genes to their human orthologs. The reference species was the human genome (hg38), and the background set was the entire human genome. To evaluate its functionality, the Biological Process (BP) category of Gene Ontology (GO) was selected. Only GO terms associated with at least three genes and with a *p*‐value less than 0.05 were considered statistically significant.

### 5.18 Plasmid Construction

To evaluate PPP3CB promoter activity and to generate a backbone for super‐enhancer assays, an approximately 2 kb PPP3CB promoter fragment was amplified by PCR from LargeWhite genomic DNA. The PCR product was separated and purified using agarose gel and then cloned into the pGL3‐Basic vector (Addgene, #212936) to create the pGL3‐PPP3CB promoter recombinant vector. Additionally, four truncated fragments (E1, E2, E3, and E4) of the LargeWhite *PPP3CB* super‐enhancer, representing different regions of the super‐enhancer, were obtained through PCR and cloned into the pGL3‐PPP3CB promoter vector to generate the super‐enhancer truncated recombinant vectors. The primers used for amplification included XhoI or HindIII restriction sites at both ends. To compare the sequence conservation/variation of the *PPP3CB* promoter among breeds, the Lean‐type group (WT) included LargeWhite and Duroc, and the Chinese indigenous group (CT) included LaiWu and JianLi. *PPP3CB* promoter sub‐fragments spanning 712–1003 bp and 712–852 bp were amplified from genomic DNA of WT and CT, respectively, and cloned into the pGL3‐Basic luciferase reporter vector to generate WT, CT, WT(712–852), and CT(712–852) constructs. For the overexpression plasmids of PPP3CB and MEF2C, the coding sequences (CDS) were amplified using specific primers with homologous arms. The amplified CDS was then inserted into the pcDNA3.1 vector (Addgene, #V790‐20) via seamless cloning, yielding pcDNA3.1‐*PPP3CB* and pcDNA3.1‐*MEF2C* constructs. All recombinant plasmids were confirmed by Sanger sequencing (Sangon, China).

### 5.19 Cell Isolation, Culture, and Transfection

Porcine skeletal muscle satellite cells (PSMSCs) were isolated from the LD muscle of 2‐week‐old (postnatal day 14) male LargeWhite and Duroc piglets using previously reported procedures [25, 92]. Muscle tissue was minced and digested with 2 mg/mL collagenase type I (Sigma‐Aldrich, USA, 0130). The digestion was stopped by adding RPMI 1640 medium containing 20% fetal bovine serum (FBS). The digested solution was then filtered using 100, 70, and 40 µm cell strainers. The isolating PBS contained 2% penicillin/streptomycin. Cells were cultured in a growth medium composed of RPMI 1640, 20% FBS, 4 ng/mL basic fibroblast growth factor, 1% chicken embryo extract, and 1% penicillin‐streptomycin at 37°C with 5% CO_2_. To induce myogenic differentiation, the cells were transferred to DMEM containing 2% horse serum (Gibco, USA) and allowed to grow until 80–90% confluent. Myogenic differentiation was achieved by plasmid transfection (4 µg) using Lipofectamine 2000 (9 µL) according to the manufacturer's protocol. *PPP3CB* siRNA oligonucleotides were designed and synthesized by AuGCT (Wuhan, China). The pig *PPP3CB* siRNA oligonucleotides (sense: UCUAAUCUUCUAAUAGCUcgGGTP; anti‐sense: GGAUGAUAUUAGAAGCUUAGATT) and MEF2C siRNA oligonucleotides (sense: CCAACAAGCUGUUCCAGUAUGTT; anti‐sense: MAUACUGGAACAGCUUG) were used in the experiment.

### 5.20 Lentiviruses‐Mediated PPP3CB and MEF2C Knockdown in Muscles

Mouse *PPP3CB*/*MEF2C* short hairpin RNA (shRNA) was inserted into the pLKO.1 lentiviral vector (Addgene, #1864) following the Addgene provided protocol. Recombinant lentiviruses (LV‐sh‐*PPP3CB*/*MEF2C*) were produced by co‐transfecting HEK293T cells with the lentiviral plasmid and packaging plasmids psPAX2 (Addgene, #12260) and pMD2.G (Addgene, #12259), following the Addgene protocol. The pLKO.1‐TRC lentiviral vector (Addgene, #10879) was used to package the control virus (LV‐sh‐NC). The experimental procedures followed the description [25, 92]. Gastrocnemius muscles of the 4‐week‐old wild‐type C57BL/6 mice were injected weekly for 4 weeks with either the negative control lentivirus (LV‐sh‐NC) or the shRNA‐targeted PPP3CB/MEF2C lentivirus (LV‐sh‐*PPP3CB*/*MEF2C*), with injection volumes of 50 µL for the first 2 weeks and 100 µL for the last 2 weeks. The lentivirus concentration for all injections was at least 1 × 10^8^ transducing units per milliliter.

### 5.21 Cell Immunofluorescence Staining

Cells were stained using previously described protocols [93]. The primary antibodies used were MyHC2a (Santa Cruz, sc‐53095; 1:100), MyHC2b (Invitrogen, 14‐6503‐80; 1:1000), MyHC1 (Santa Cruz, sc‐53090; 1:50), and MyHC2x (DSHB, 6H1; 1:100). DAPI was used to stain the cell nuclei, and fluorescence images were captured using a fluorescence microscope (Olympus, DP80).

### 5.22 Luciferase Reporter Assay

pSMSCs were transfected with *PPP3CB* promoter recombinant vectors, super‐enhancer truncated recombinant vectors, and pcDNA3.1‐MEF2C vector using Lipofectamine 2000 (Invitrogen, #11668027). After 2 days of differentiation in a 24‐well plate, the cells were washed with PBS and lysed with 100 µL lysis buffer. The collected cells were analyzed for regulatory region activity using a dual‐luciferase reporter assay system (Promega, USA, E2920). Luciferase activity was measured using a PerkinElmer 2030 Multilabel Reader (PerkinElmer). To ensure accurate transfection efficiency, the cells were co‐transfected with 0.04 µg of the pRL‐TK (Promega, #E2241).

### 5.23 CUT&Tag qPCR

CUT&Tag qPCR was performed using the Hyperactive Universal CUT&Tag Assay for Illumina Pro Kit (Vazyme, TD904, China) following the manufacturer's protocol. Purified DNA was used for qRT‐PCR analysis. The primers used for CUT&Tag qPCR detection are listed in Table S4. The MEF2C antibody and an IgG antibody (Proteintech, 30000‐0‐AP, China) were used in the experiment.

### 5.24 3C‐PCR/qPCR

3C analysis was conducted as previously described in the literature [94]. Briefly, chromatin was crosslinked using 1% formaldehyde, and nuclei were extracted using Nonidet P‐40 (Beyotime, ST2045). DNA was digested with 800 units of MboI (NEB, R0147V) and ligated in 8 mL of 1× ligation buffer. The 3C products were extracted using phenol/chloroform, ethanol‐precipitated, quantified by UV spectrophotometry, and analyzed via PCR. The PCR conditions included initial denaturation at 95°C for 5 min, followed by 35 cycles of 95°C for 45 s, 60°C for 45 s, 72°C for 60 s, and a final extension at 72°C for 5 min. Primers targeting the *PPP3CB* promoter and super‐enhancer core functional region were designed using Primer3 [95]. The design parameters included a primer length of 26 nucleotides (range 24–28), optimal melting temperature (Tm) of 62°C (range 60–64), and amplicon size of 200 base pairs, with the restriction site positioned midway between the primers. Undigested, non‐ligated genomic DNA served as a template control, and GAPDH amplification was used as an internal control. For 3C‐qPCR, the ligated 3C products were quantitatively analyzed using SYBR Green‐based PCR, with GAPDH primers serving as normalization controls. Primer sequences are available in Table S4.

### 5.25 CRISPRi Assay

CRISPRi‐mediated enhancer repression was performed using the pX330a Cas9‐KRAB vector (Addgene, #92361) [96]. sgRNAs (Table S4) were designed using CRISPOR [97]. A primer pair (20 µL at 100 µmol/L) and 80 µL of H_2_O were heated to 95°C for 5 min, then cooled to room temperature to form a dimer. The pX330a Cas9‐KRAB vector was digested with Bbs I (NEB, R0539V) and purified by gel extraction. The vector was then ligated with the dimer using T4 DNA ligase (NEB, M0202V) at 16°C overnight. The recombinant vectors were confirmed and transfected into PSMSCs at 50%–60% confluence, grown in 6‐well plates, and collected after 24 h of incubation.

### 5.26 Statistical Analyses

All data were presented as mean ± standard deviation (SD). Sample sizes (n) were indicated in the legends. Statistical analyses were performed using GraphPad Prism and R (v4.1). Data distribution was assessed prior to hypothesis testing. For comparisons between two groups, a two‐sided Student's *t*‐test (paired or unpaired as appropriate) was used for approximately normally distributed data; otherwise, the Wilcoxon rank‐sum test was used for unpaired data (and the Wilcoxon signed‐rank test for paired data, when applicable). For analyses involving three or more groups, one‐way ANOVA (aov) followed by Tukey's HSD was used when model assumptions were met; otherwise, nonparametric Kruskal–Wallis tests followed by appropriate post hoc multiple comparisons were applied. A *p* value < 0.05 was considered statistically significant, and significance levels were indicated at *p* < 0.05, *p* < 0.01, and *p* < 0.001.

## Author Contributions

D.X. and M.Z. conceived and designed this study. S.Z., H.W., M.L., and K.W. performed the experiments. S.Z., H.W., M.L., K.W., S.H., Z.A., and L.P. conducted the data analysis and prepared figures and tables. D.X., M.L., and S.Z. wrote the manuscript. All authors reviewed and approved the manuscript.

## Funding

National Key Research and Development Program of China (Grant No.2021YFD1301201), Major Project of Agricultural Biological Breeding of China (2023ZD0404702), Science and Technology Project of Xinjiang Production and Construction Corps (Grant No.2022AB012), Agricultural Science Innovation Foundation of Hubei Province (Grant No.2025–620‐000–001‐026), State Key Development Program for Basic Research of China (2014CB138504), Research Project of Huazhong Agricultural University, China.

## Conflicts of Interest

The authors declare no conflict of interest

## Supporting information




**Supporting File 1**: advs75292‐sup‐0001‐SuppMat.docx.

**Supporting File 2**: advs75292‐sup‐0002‐TableS1‐S4.zip.

## Data Availability

The ChIP‐seq (H3K27ac and H3K4me3) and ATAC‐seq data for LargeWhite and Duroc pigs analyzed in this study were obtained from the NCBI Sequence Read Archive (SRA) under BioProject PRJNA597497. Hi‐C data for LargeWhite pigs were downloaded from GEO under accession number GSE143288. Whole‐genome sequencing (WGS) data were retrieved from the NCBI SRA (PRJNA260763 and PRJNA213179) and the GSA at the National Genomics Data Center (PRJCA030193). The sequencing data generated in this study have been deposited in the NCBI BioProject database under accession number PRJNA1266095. Details of the public datasets are provided in Supplementary Table 1.
