# Supplementary material for: Multi‐Omics Insights Into the Mechanisms of Early Muscle Fiber Difference and Transformation Between Lean‐Type and Chinese Indigenous Pigs
Source: Adv Sci (Weinh). 2026 Apr 17;13(38):e23959. doi: 10.1002/advs.202523959 (PMC13334937; doi:10.1002/advs.202523959)

Supporting Information

Multi-omics Insights into the Mechanisms of Early Muscle Fiber Difference and Transformation between Lean-type and Chinese Indigenous Pigs

*Shuailong Zheng, Hainan Wu, Min Liu, Kunpeng Wu, Shuntao Huang, Zulfiqar Ahmed, Lenox Pius, Mengjin Zhu* and Dequan Xu**

**Supplementary Figures 1–18**

**Figure S1.** Histological analysis of skeletal muscles in Chinese indigenous and lean-type pigs.

**Figure S2.** Data quality summary for RNA-seq, ATAC-seq, and histone ChIP-seq (H3K4me3 and H3K27ac) in lean-type and Chinese indigenous pigs.

**Figure S3.** Mapping of OCRs and epigenomic marks.

**Figure S4.** Analysis of transcription factor motifs in different OCRs.

**Figure S5.** Dynamic OCRs and histone modifications are associated with differences in gene expression between Chinese indigenous and lean-type pigs.

**Figure S6.** Identification of regulatory elements in Chinese indigenous and lean-type pigs.

**Figure S7.** Identification of genetic variants in Chinese indigenous and lean-type pigs.

**Figure S8.** Summary of Hi-C data for LargeWhite and LaiWu pigs.

**Figure S9.** Characteristics of compartments A/B in Chinese indigenous and lean-type pigs.

**Figure S10.** Compartment switching in Chinese indigenous and lean-type pigs.

**Figure S11.** Characteristics of TADs in LargeWhite and LaiWu pigs.

**Figure S12.** Basic features of PEIs and loops.

**Figure S13.** Differences in *PPP3CB* expression in Chinese indigenous and lean-type pigs.

**Figure S14.** The interaction between the *PPP3CB* promoter and the super enhancer requires MEF2C.

**Figure S15.** Cross-breed evidence for a lean-type–specific SE and CRISPRi-based functional validation.

**Figure S16.** Summary of *PPP3CB* perturbation by knockdown and overexpression.

**Figure S17.** Analysis of the amino acid sequence conservation of PPP3CB and MEF2C, and the *PPP3CB* promoter sequence in Chinese indigenous pigs and lean-type pigs.

**Figure S18.** Lentivirus-mediated *MEF2C* knockdown in muscles significantly increased muscle mass and the proportion of glycolytic muscle fibers.


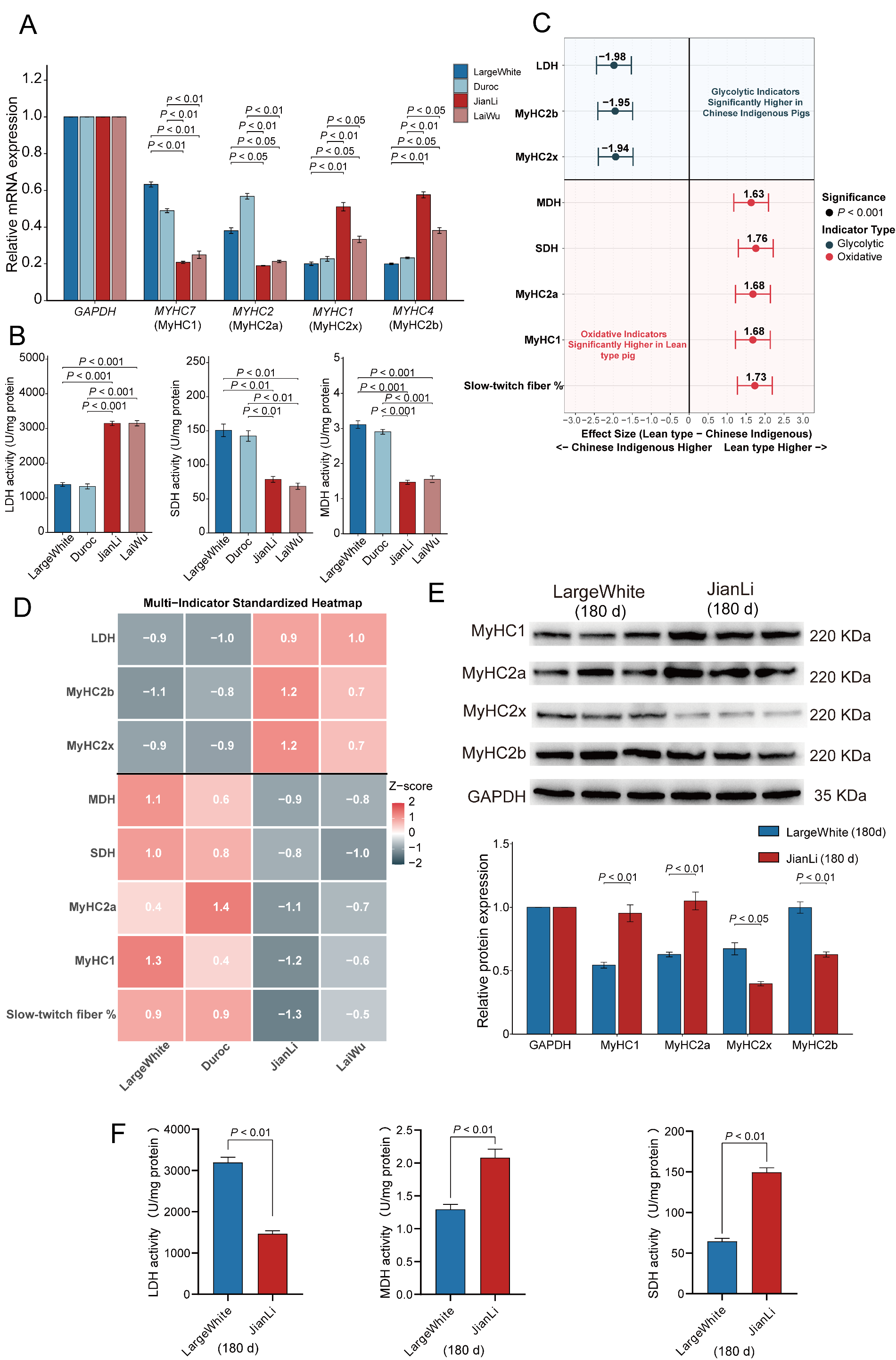


**Figure S1.** Histological analysis of skeletal muscles in Chinese indigenous and lean-type pigs. A) The mRNA expression levels of MyHC1, MyHC2a, MyHC2b, and MyHC2x in two-week-old lean-type pigs and Chinese indigenous pigs were quantified by qPCR (n = 3 per breed). B) Activity of SDH, MDH, and LDH in two-week-old lean-type and Chinese indigenous pigs (n = 3 per breed). C) Mixed-effects model estimates of breed-type effects (Lean − Chinese indigenous) with 95% confidence intervals across indicators. D) Heatmap summary of mixed-effects model estimates (Lean − Chinese indigenous) across muscle fiber and enzyme activity indicators. E) Western blot analysis of different muscle fiber type protein levels in 180-day-old LargeWhite and JianLi pigs (n = 3 per breed). F) Activity of SDH, MDH, and LDH in LargeWhite and JianLi pigs (180 d) (n = 3 per breed). Data are shown as the mean ± standard deviation (SD). *P* values were calculated using Student's t-test.


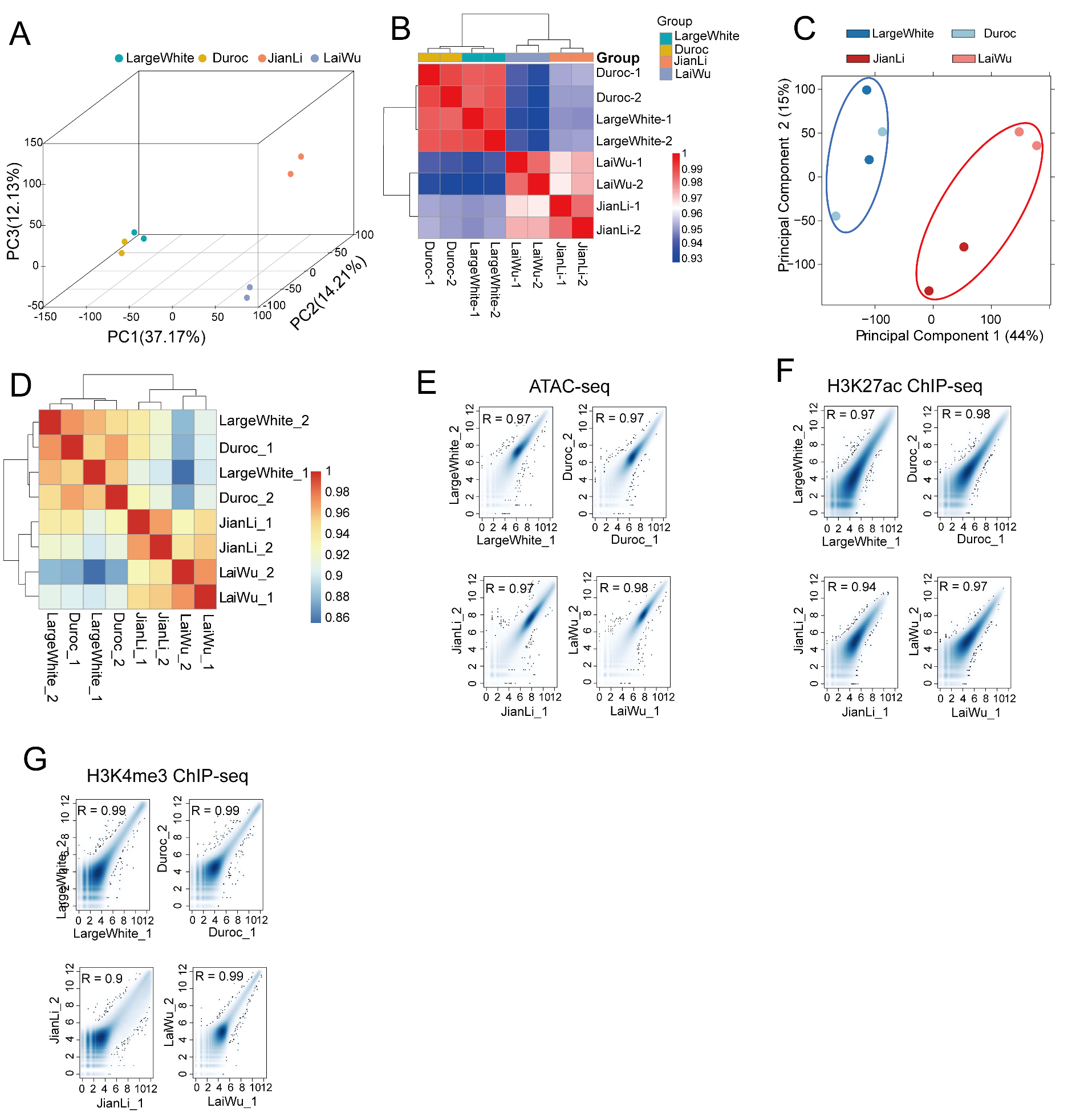


**Figure S2.** Data quality summary for RNA-seq, ATAC-seq, and histone ChIP-seq (H3K4me3 and H3K27ac) in lean-type and Chinese indigenous pigs**.** A) 3D PCA analysis of RNA-seq data of Chinese indigenous and lean-type pig *longissimus dorsi*. Each breed contained two biological replicates. B) Correlation heatmap of RNA-seq data from Chinese indigenous and lean-type pigs. C) PCA analysis of ATAC-seq data from Chinese indigenous and lean-type pigs. D) Correlation heatmap of ATAC-seq data from Chinese indigenous and lean-type pigs. E-G) Correlation analysis of read intensity between replicates for ATAC-seq and histone ChIP-seq (H3K4me3 and H3K27ac). The number per 10 kb was used for read intensity evaluation. Pearson's correlation coefficients (R) are shown in the upper left corner.


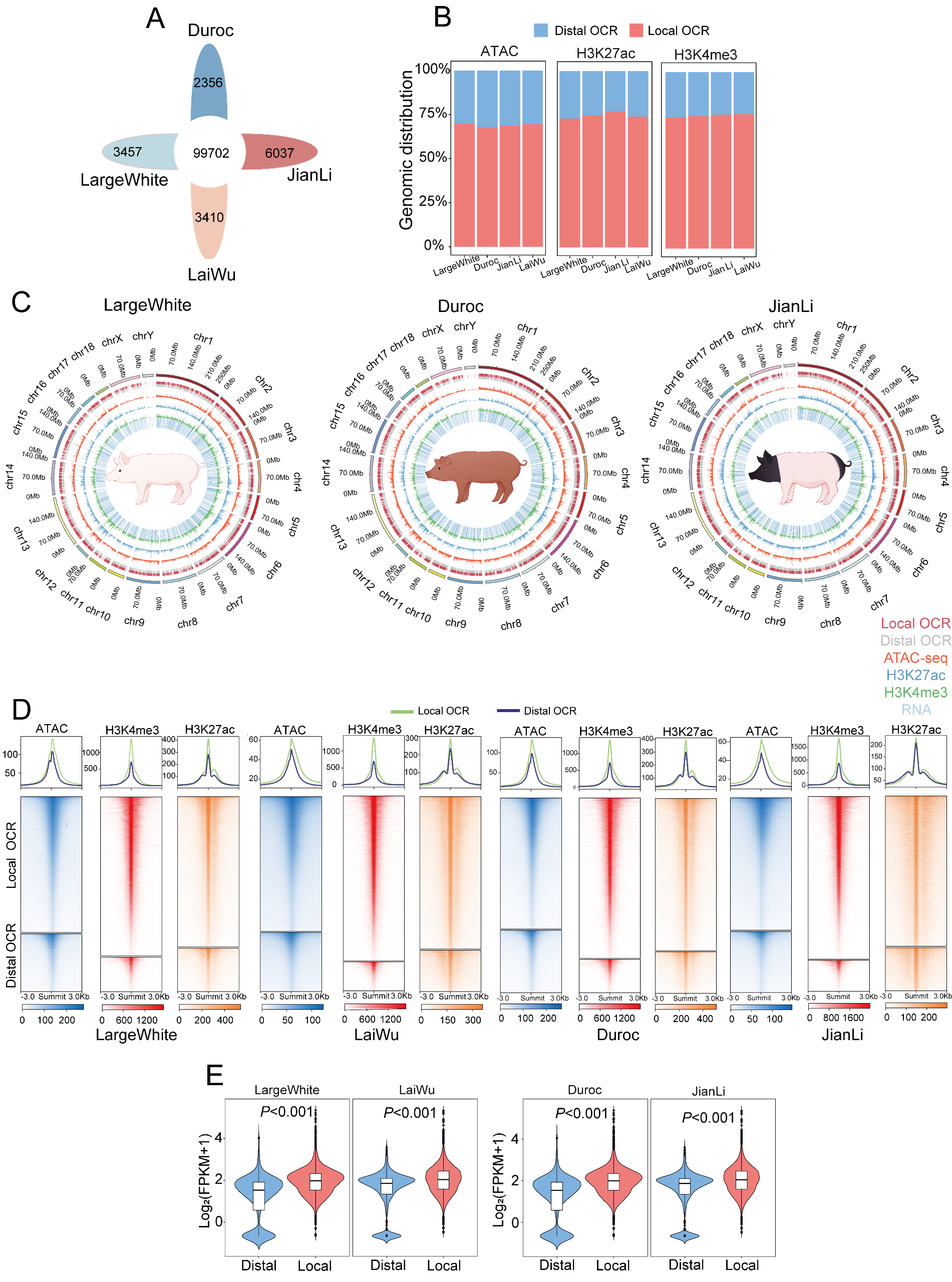


**Figure S3.** Mapping of OCRs and epigenomic marks. A) ATAC-seq peak petal plot of Chinese indigenous and lean-type pigs. B) Distribution of LoOCRs, dOCRs, and histone modifications (H3K4me3 and H3K27ac) in lean-type and Chinese indigenous pigs. C) Circos plot of gene expression (RNA-seq), chromatin accessibility (ATAC-seq), H3K4me3, H3K27ac, LoOCRs, and dOCRs across the genome in LargeWhite, Duroc, and JianLi pigs. D) Epigenome profiles at LoOCRs and dOCRs centered on OCR summits in Chinese indigenous and lean-type pigs. The regions ± 3 kb from the OCR summits are shown. E) Compared to genes with only distal OCR, genes with local OCR exhibited significantly higher expression levels in Chinese indigenous and lean-type pigs. *P* values were calculated using the two-sided Wilcoxon rank-sum test in (E).


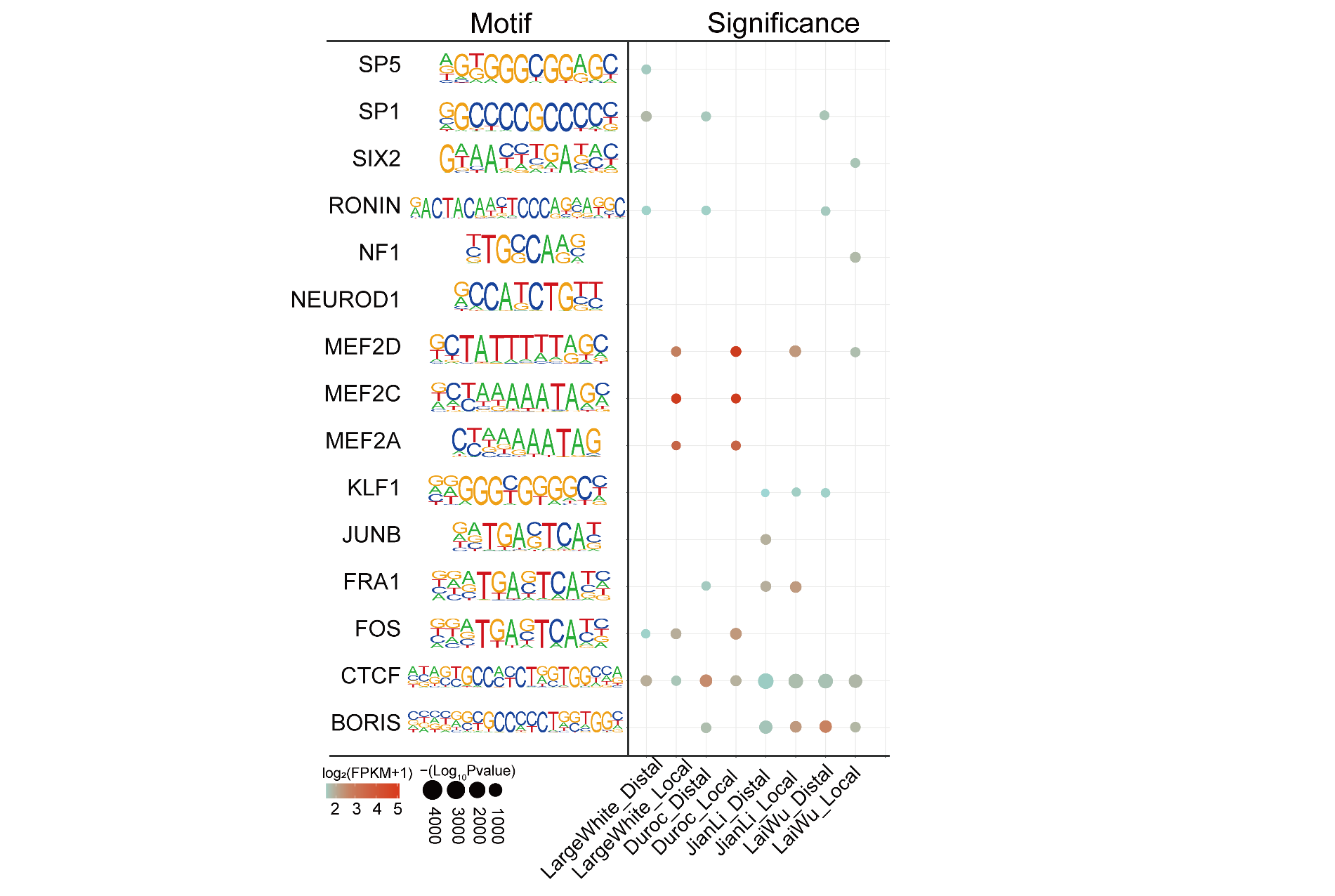


**Figure S4.** Analysis of transcription factor motifs in different OCRs. TF motifs abundant in LoOCRs and dOCRs of lean-type and Chinese indigenous pigs.


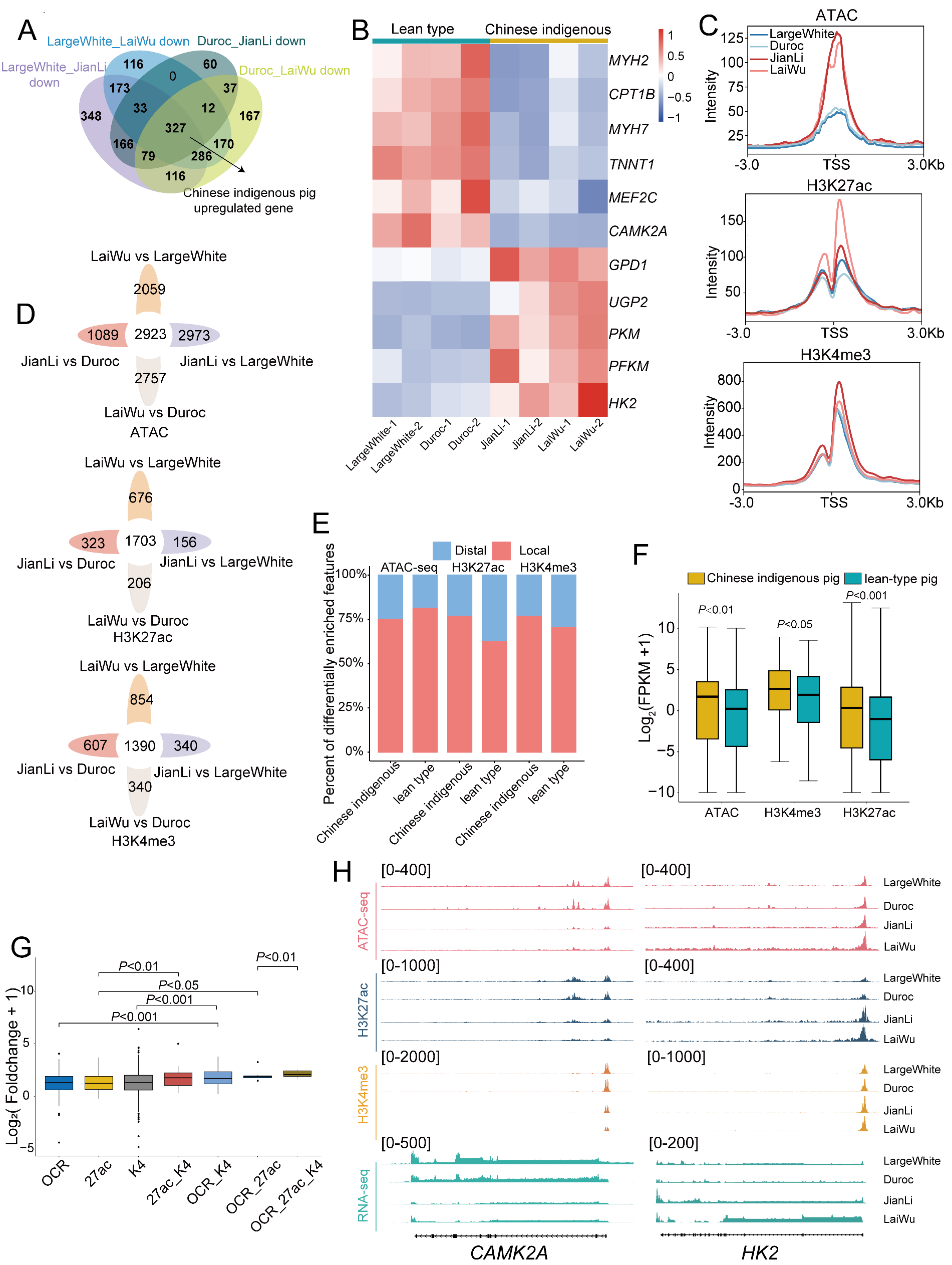


**Figure S5.** Dynamic OCRs and histone modifications are associated with differences in gene expression between Chinese indigenous and lean-type pigs. A) Overlap of genes upregulated in Chinese indigenous pigs relative to lean-type breeds. B) Heatmap of muscle fiber-type associated gene expression. C) Average signal intensities of ATAC-seq, H3K27ac, and H3K4me3 within a 3 kb window around the Transcription Start Site (TSS) of upregulated genes in Chinese indigenous pigs. D) Flower plot of differential ATAC-seq, H3K4me3, and H3K27ac peaks between Chinese indigenous pigs and lean-type pigs. E) Enrichment percentages of ATAC-seq signals, H3K27ac, and H3K4me3 modifications in local and distal OCRs of Chinese indigenous and lean-type pigs. F) Gene expression levels of genes enriched in ATAC-seq, H3K27ac, and H3K4me3 modifications in Chinese indigenous pig. G) Fold change (FC) in the expression of Chinese indigenous pig-enriched gene sets associated with single or combined chromatin state changes. K4 denotes H3K4me3; 27ac denotes H3K27ac. H) Examples of *CAMK2A* (upregulated in lean-type pig), and *HK2* (upregulated in Chinese indigenous pig), showing differences in their expression and chromatin states in Chinese indigenous and lean-type pig. *P* values were calculated using the two-sided Wilcoxon rank-sum test in (F,G).


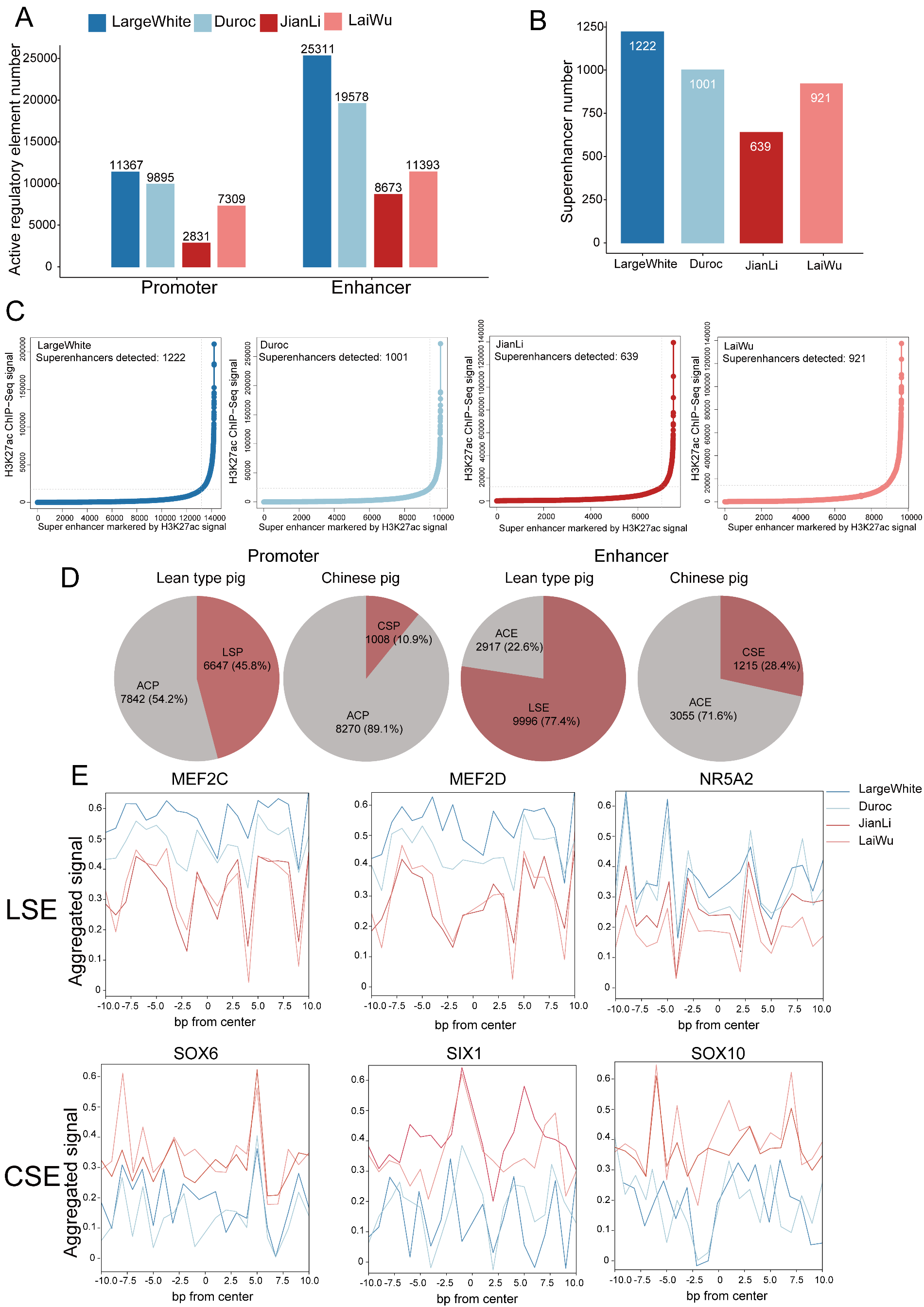


**Figure S6.** Identification of regulatory elements in Chinese indigenous and lean-type pigs. A) Statistical analysis of the number of active promoters and enhancers in Chinese indigenous and lean-type pigs. B-C) Identification and statistical analysis of super-enhancers in Chinese indigenous and lean-type pigs. D) Statistical analysis of conserved and breed-specific active enhancers and promoters in Chinese indigenous and lean-type pigs. ACP, conserved active promoters between lean-type pigs and Chinese indigenous pigs; ACE, conserved active enhancers between lean-type pigs and Chinese indigenous pigs; LSP, active promoters specific to lean-type pigs; LSE, active enhancers specific to lean-type pigs; CSP, active promoters specific to Chinese indigenous pigs; CSE, active enhancers specific to Chinese indigenous pigs. E) Aggregate TF footprint plots for MEF2C, MEF2D, NR5A2, SOX6, SIX1, and SOX10 in LSE and CSE.


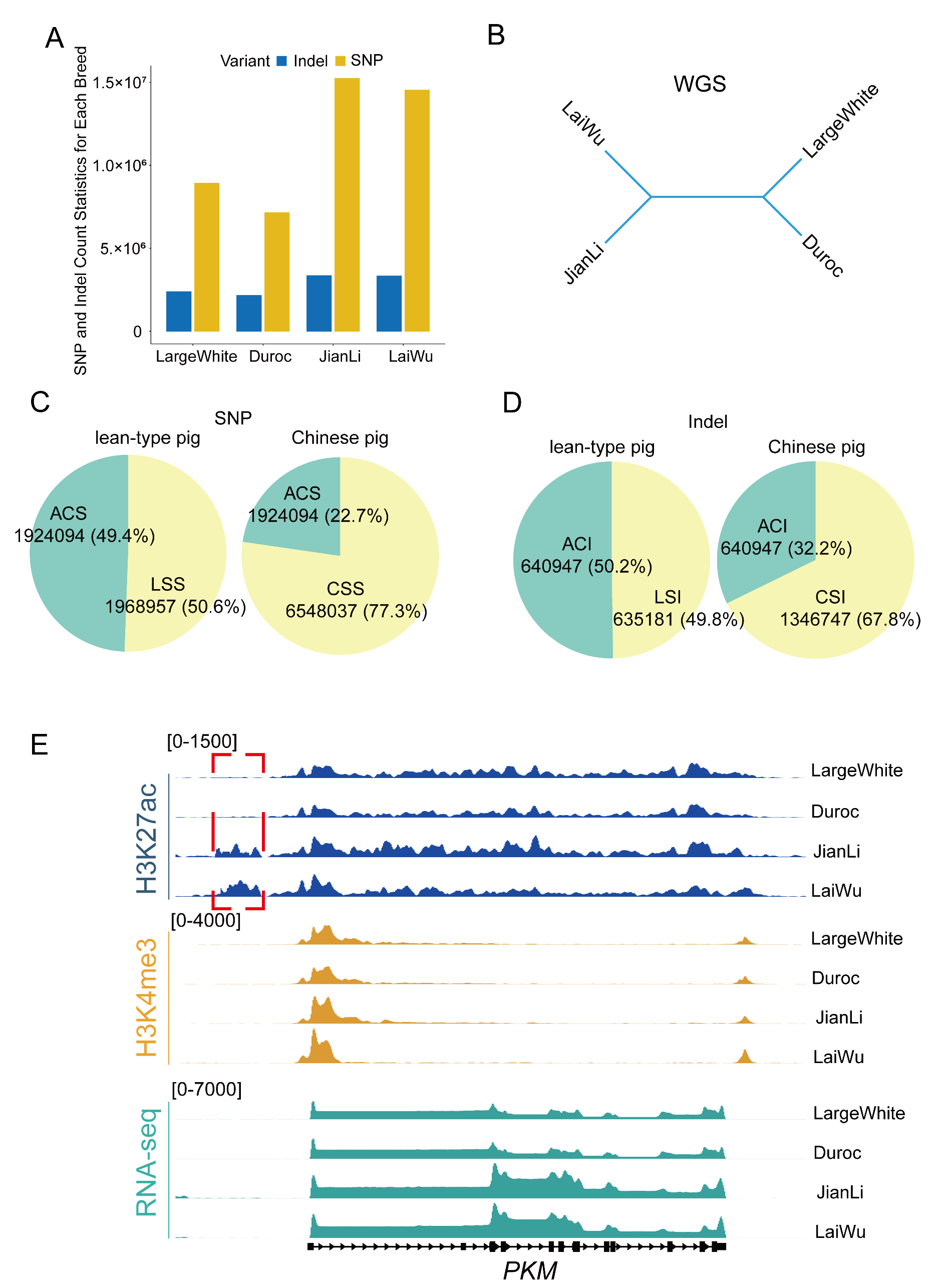


**Figure S7.** Identification of genetic variants in Chinese indigenous and lean-type pigs. A) SNP and Indel count statistics for each breed. B) The phylogenetic tree was constructed using the neighbor-joining method, showing the evolution of genome sequences based on whole-genome sequencing data. C-D) Statistical analysis of conserved and breed-specific SNPs and Indels in Chinese indigenous and lean-type pigs. ACS, shared SNPs between lean-type pigs and Chinese indigenous pigs; ACI, shared Indels between lean-type pigs and Chinese indigenous pigs; LSS, SNPs specific to lean-type pigs; LSI, Indels specific to lean-type pigs; CSS, SNPs specific to Chinese indigenous pigs; CSI, Indels specific to Chinese indigenous pigs. E) A breed-specific enhancer is located upstream of *PKM* in Chinese indigenous pigs, indicated by the red curve.


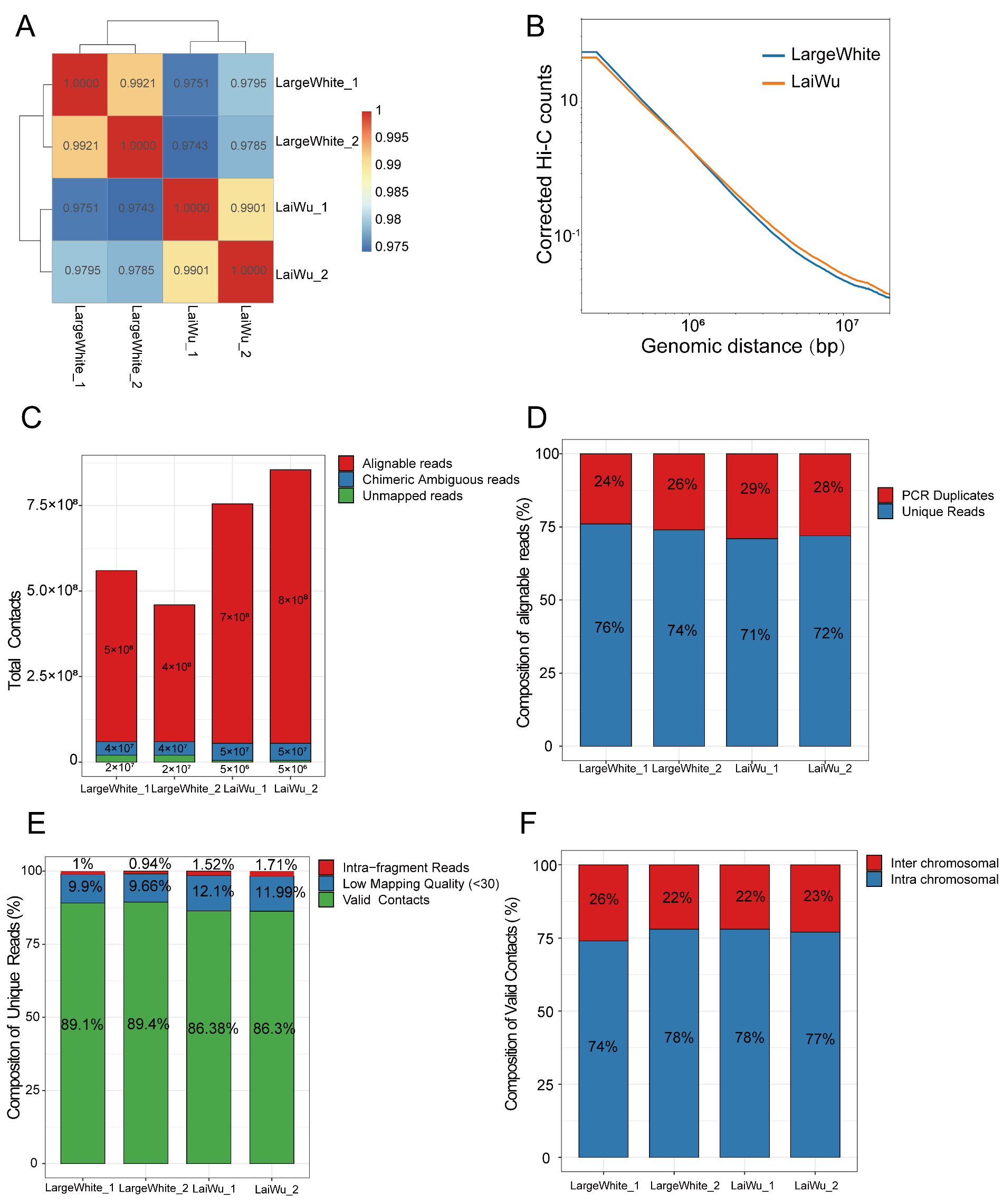


**Figure S8.** Summary of Hi-C data for LargeWhite and LaiWu pigs. A) Correlation heatmap of Hi-C data of LaiWu and LargeWhite *longissimus dorsi*. B) Contact frequency as a function of genomic distance. C) The summary of Total contacts. D) Composition of alignable reads. E) Composition of unique reads. F) Composition of Valid contacts.


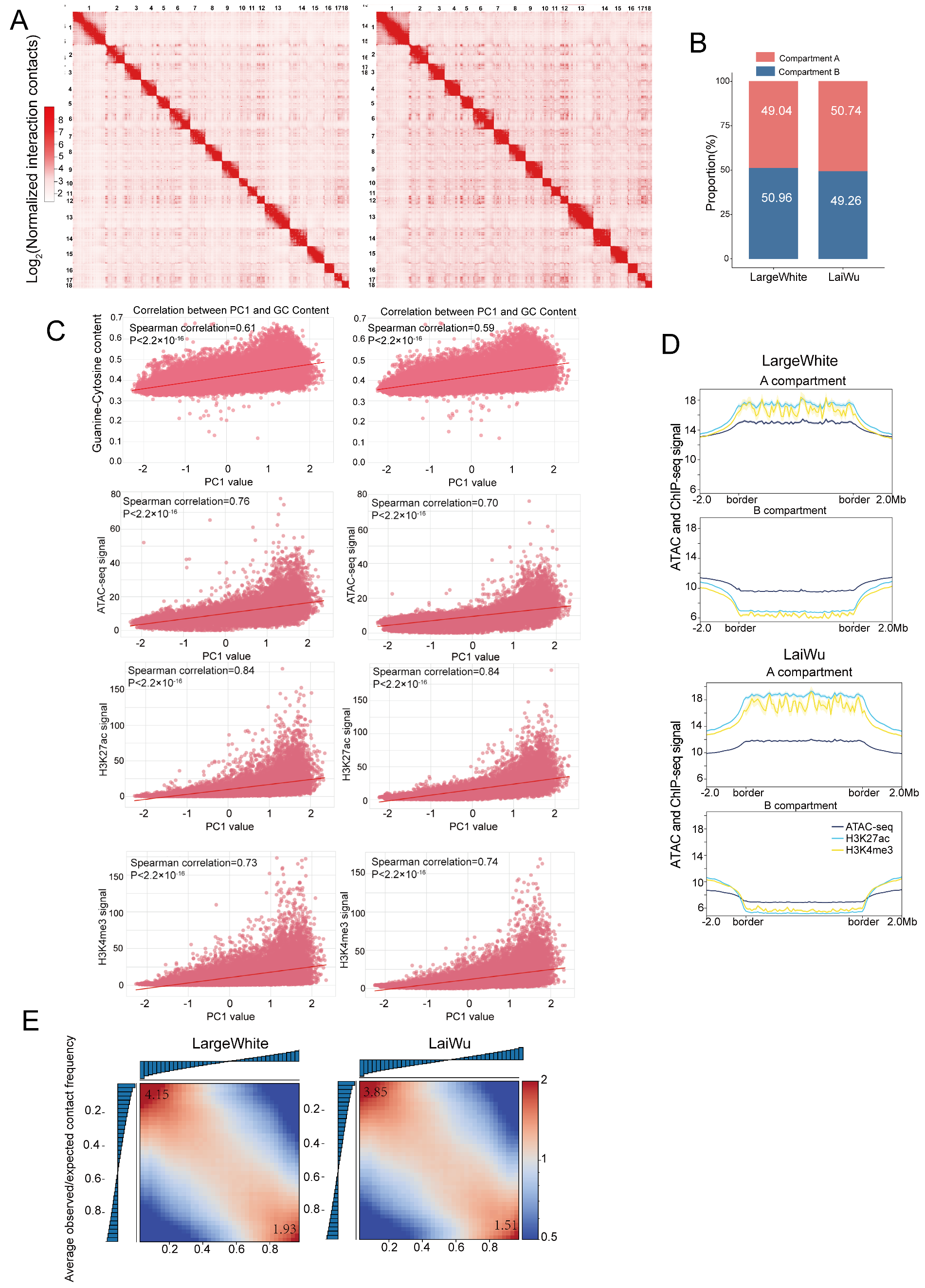


**Figure S9.** Characteristics of compartments A/B in Chinese indigenous and lean-type pigs. A) Example of observed/expected contact matrices between 18 autosomes in LargeWhite and LaiWu pigs. B) Proportions of A and B compartments in LargeWhite and LaiWu pigs. C) Scatter plots showing the correlation between PC1 values and guanine-cytosine (GC) content, ATAC-seq signals, H3K27ac signals, and H3K4me3 signals in LargeWhite and LaiWu pigs. D) Aggregated ATAC-seq, H3K27ac, and H3K4me3 signal intensities in A and B compartments in LargeWhite and LaiWu pigs. E) Average compartment strength in LargeWhite and LaiWu pigs.


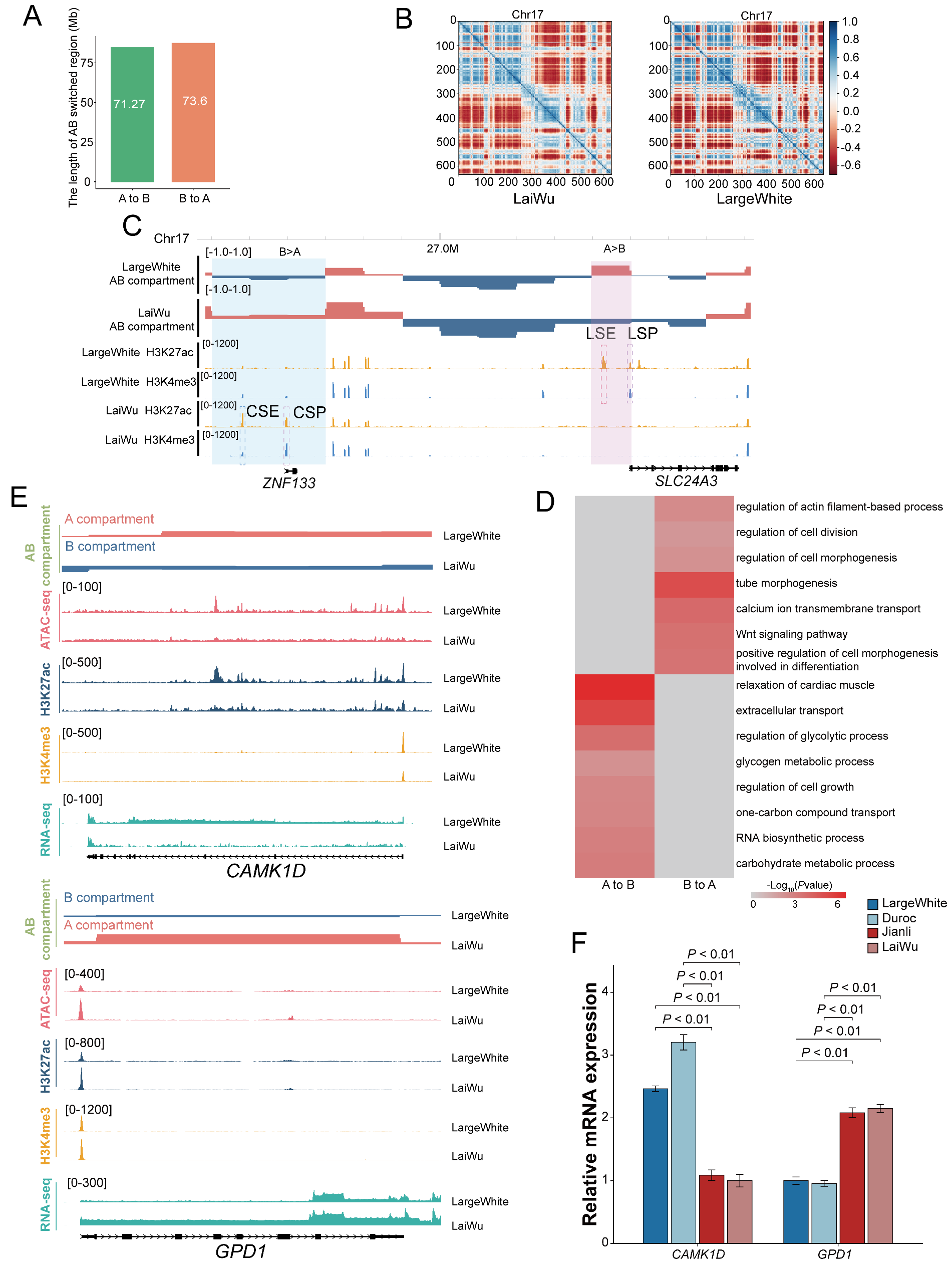


**Figure** **S10.** Compartment switching in Chinese indigenous and lean-type pigs. A) Cumulative lengths of switched compartments. B) Observed/expected normalized Hi-C contact maps for chromosome 17 at 20-kb resolution. C) Zoomed-in view of a chromosome 17 region showing A/B compartment switching (PC1) together with breed-specific active enhancers/promoters (CSE/CSP and LSE/LSP). D) Significantly enriched GO biological process (GO-BP) terms for genes located in compartment transitions (A-to-B and B-to-A). E) Representative loci showing compartment switching at *CAMK1D* and *GPD1* in LargeWhite and LaiWu pigs. F) qPCR analysis of *CAMK1D* and *GPD1* expression during A/B compartment switching (n = 3 per group). Data are presented as mean ± SD; *P* values were calculated using an unpaired two-sided Student’s t-test.


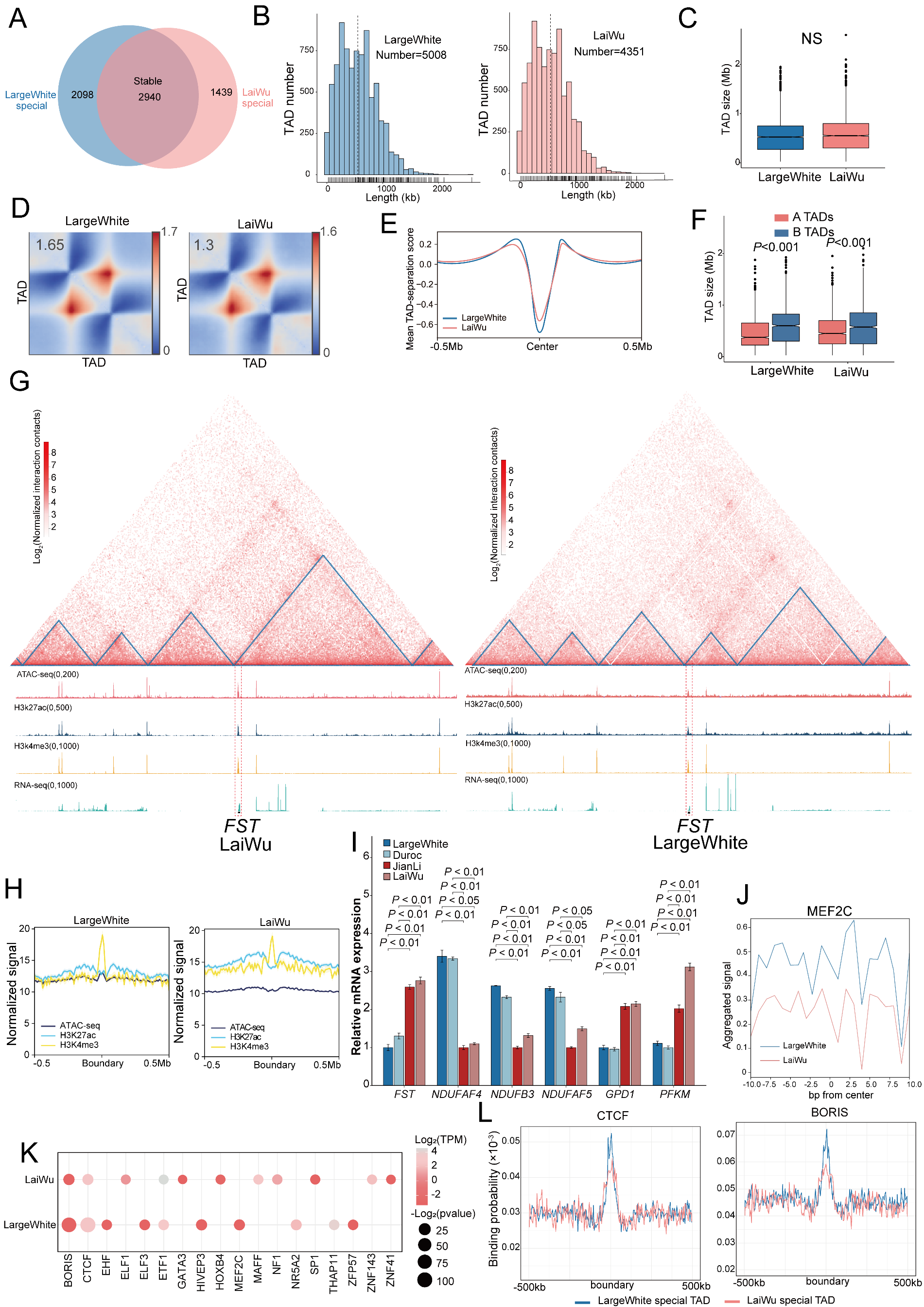


**Figure S11.** Characteristics of TADs in LargeWhite and LaiWu pigs. A) Overlap of TAD boundaries between LargeWhite and LaiWu pigs. B) Frequency distribution of TAD sizes. C) Boxplots of TAD sizes in LargeWhite and LaiWu pigs. D) Average TAD contact patterns illustrating intra-TAD and inter-TAD interactions in LargeWhite and LaiWu pigs. E) TAD boundary strength profiles in LargeWhite and LaiWu pigs. F) TAD size comparison between compartments A and B. G) Influence of TAD boundary variations on the expression of the FST gene in LargeWhite and LaiWu pigs. H) Distribution of ATAC-seq, H3K27ac, and H3K4me3 signals at the TAD boundaries. I) qPCR analysis of gene expression associated with TAD boundary changes (n = 3 per group). Data are presented as mean ± SD; *P* values were calculated using an unpaired two-sided Student’s t-test. J) Aggregate TF footprint plots for MEF2C at special TAD boundaries in LargeWhite and LaiWu pigs. K) Transcription factor motif analysis of special TAD boundaries in LargeWhite and LaiWu pigs. L) Enrichment of CTCF and BORIS at the TAD boundaries. ns indicates statistical non-significance. *P* values were calculated using the two-sided Wilcoxon rank-sum test in (C, F). NS indicates no significant difference.

**
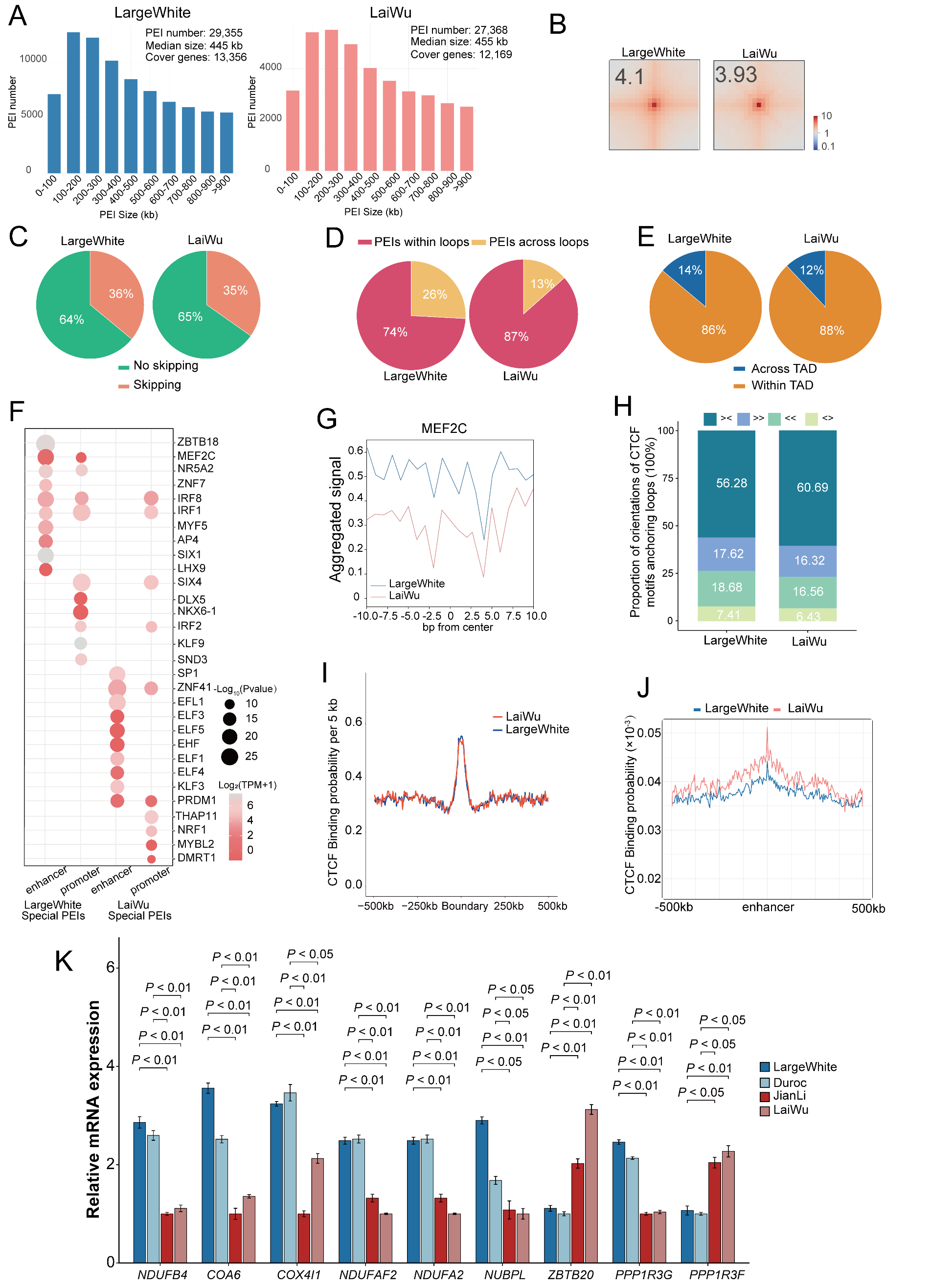
**

**Figure S12.** Basic features of PEIs and loops. A) Size distribution of PEIs in LargeWhite and LaiWu pigs. B) The average loop strength of the LargeWhite and LaiWu pigs. C) Ratio of enhancers interacting with the nearest promoters (no skipping) or skipping at least one promoter. D) Proportion of PEIs located within or across loops. E) Proportion of PEIs located within or across TADs. F) Analysis of transcription factor motifs within the promoters and enhancers of the breed-specific PEIs in LargeWhite and LaiWu pigs. G) Aggregate TF footprint plots for MEF2C at special PEIs in LargeWhite and LaiWu pig. H) Proportion of orientations of CTCF motifs anchoring loops. I) Enrichment of CTCF motifs at TAD boundaries. J) Enrichment of CTCF motifs at the enhancer region. K) qPCR analysis of expression levels of genes associated with rewired PEIs (n = 3 per group). Data are presented as mean ± SD; *P* values were calculated using an unpaired two-sided Student’s t-test.


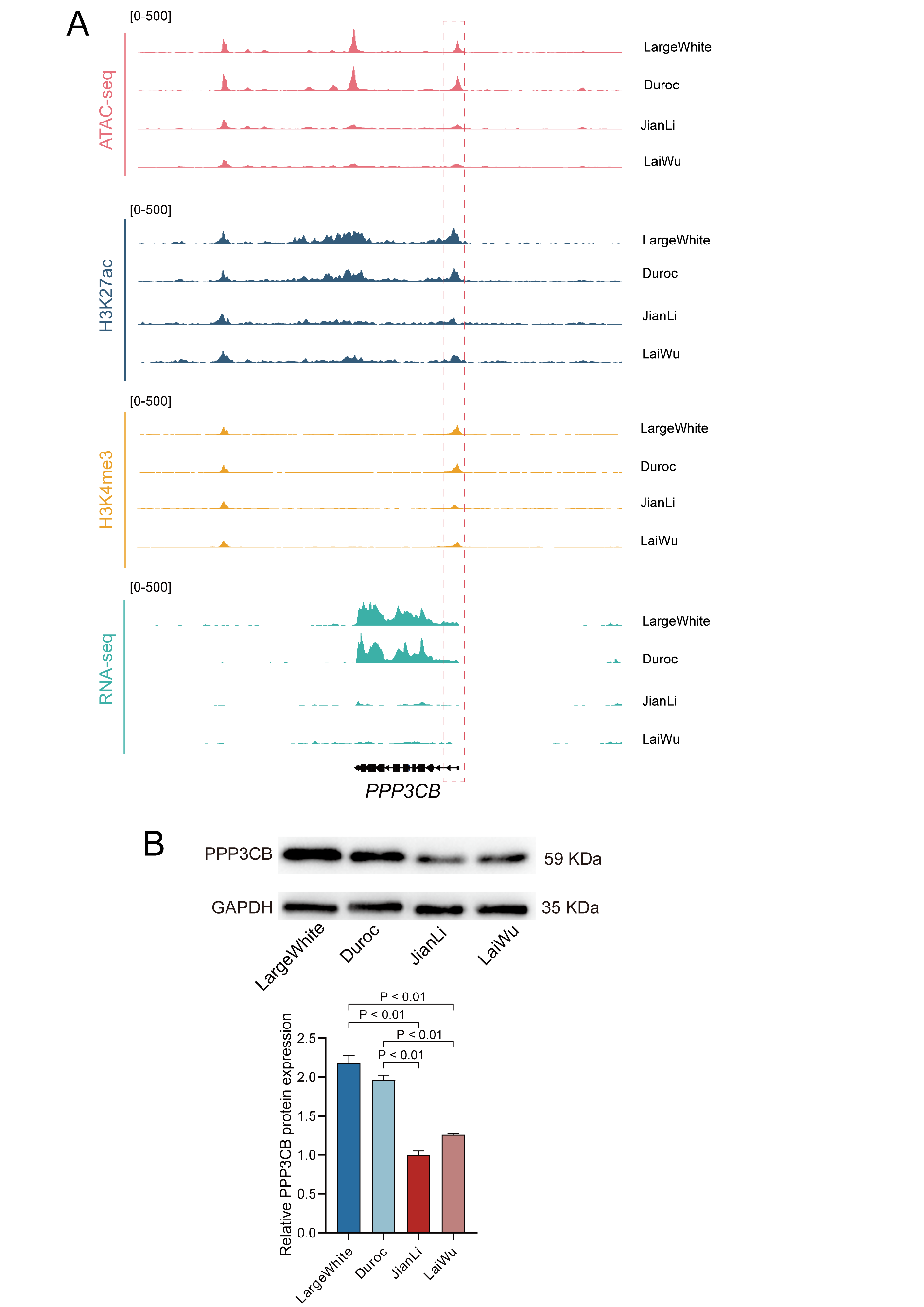


**Figure S13.** Differences in *PPP3CB* expression in Chinese indigenous and lean-type pigs. A) Multi-omics visualization of *PPP3CB* in Chinese indigenous and lean-type pigs. B) Protein expression levels of PPP3CB in different pig breeds (n = 3 per group). Data are shown as the mean ± standard deviation (SD), and *P* values were calculated using a two-sided Student’s t-test.


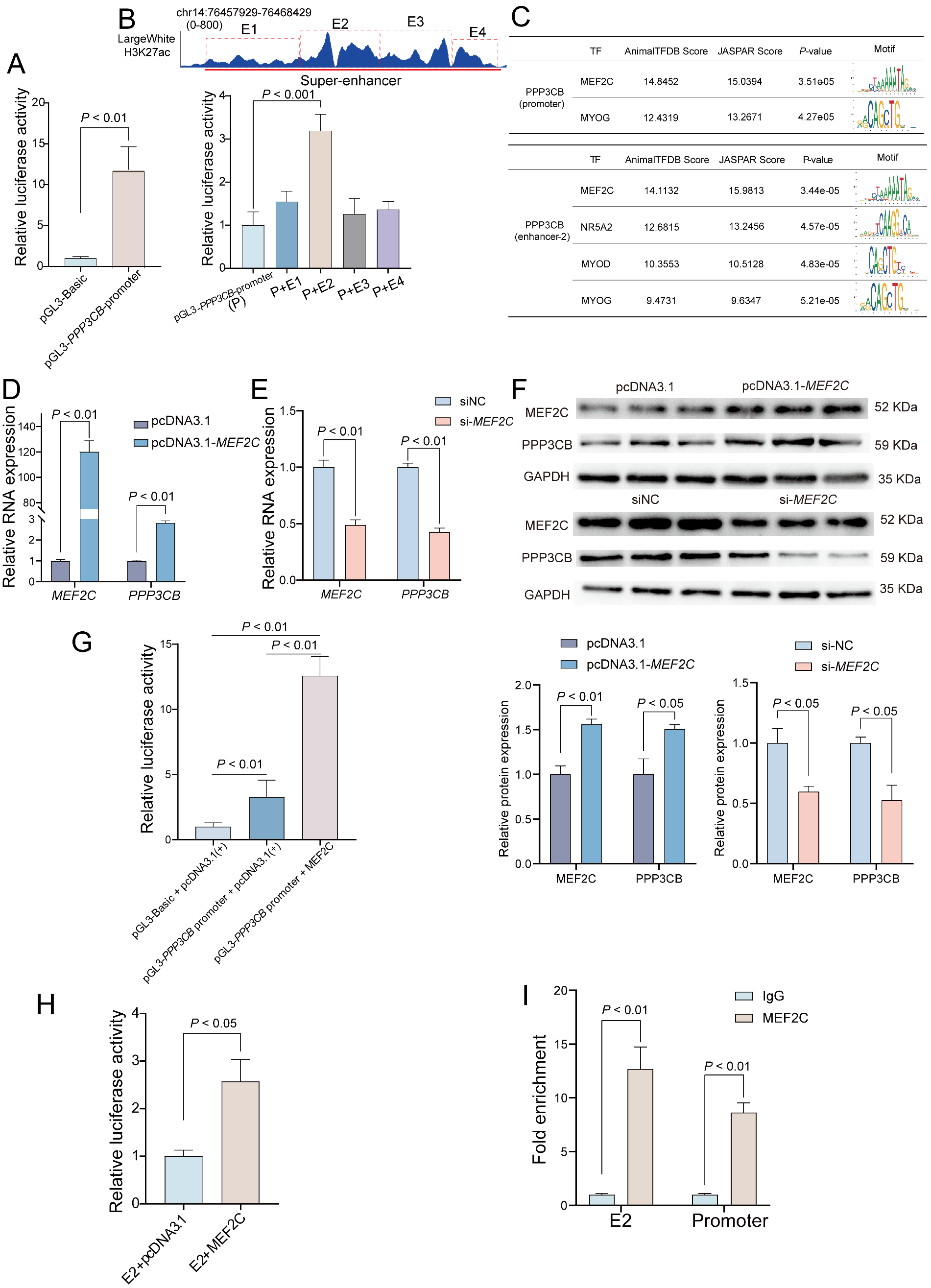


**Figure S14.** The interaction between the *PPP3CB* promoter and the super enhancer requires MEF2C. A) Identification of *PPP3CB* promoter activity (n = 3 per group). B) Identification of the core functional domain of *PPP3CB* super-enhancer (n = 3 per group). P, pGL3-*PPP3CB*-promoter; P+E1, pGL3-*PPP3CB*-promoter+E1; P+E2, pGL3-*PPP3CB*-promoter+E2; P+E3, pGL3-*PPP3CB*-promoter+E3; P+E4, pGL3-*PPP3CB*-promoter+E4. C) Prediction of the core regulatory region of the *PPP3CB* super-enhancer and its promoter transcription factor binding sites (using the AnimalTFDB and JASPAR databases). D-E) Overexpression and interference of *MEF2C*; qPCR detection of *MEF2C* and *PPP3CB* mRNA changes (n = 3 per group). F) Western blot analysis of MEF2C and PPP3CB following *MEF2C* overexpression or knockdown (n = 3 per group). G) Dual-luciferase reporter assay verifying the interaction between the *PPP3CB* promoter region and transcription factor MEF2C (n = 3 per group). H) Dual-luciferase reporter assay verifying the interaction between *PPP3CB* super-enhancer core functional domain and transcription factor MEF2C (n = 3 per group). I) CUT&Tag qPCR validation of the interaction between the *PPP3CB* super-enhancer core functional domain and promoter region with MEF2C (n = 3 per group). Data are presented as mean ± SD; *P* values were calculated using an unpaired two-sided Student’s t-test.


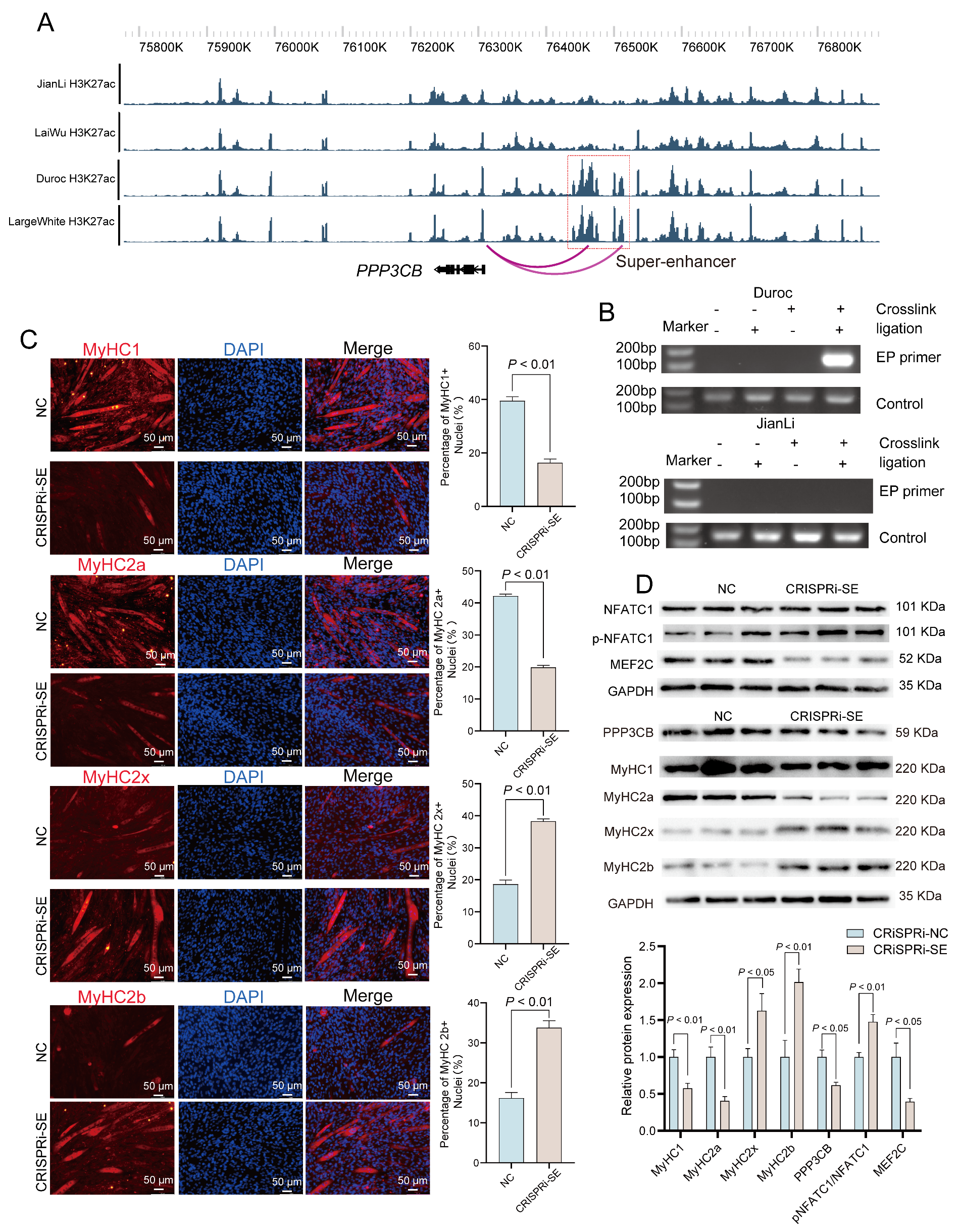


**Figure S15.** Cross-breed evidence for a lean-type–specific SE and CRISPRi-based functional validation. A) IGV tracks showing the super-enhancer (SE) signal in lean-type breeds (LargeWhite and Duroc) but not detected in Chinese indigenous breeds (JianLi and LaiWu). B) 3C analysis of SE-promoter interaction in Duroc and JianLi. C) CRISPRi-mediated repression of the SE and quantification of the proportion of myotubes positive for different muscle fiber-type markers in Duroc myotubes. D) Western blot analysis of fiber-type marker proteins and pathway-related proteins in Duroc myotubes following CRISPRi-mediated repression of the SE. Data are presented as mean ± SD; *P* values were calculated using an unpaired two-sided Student’s t-test.


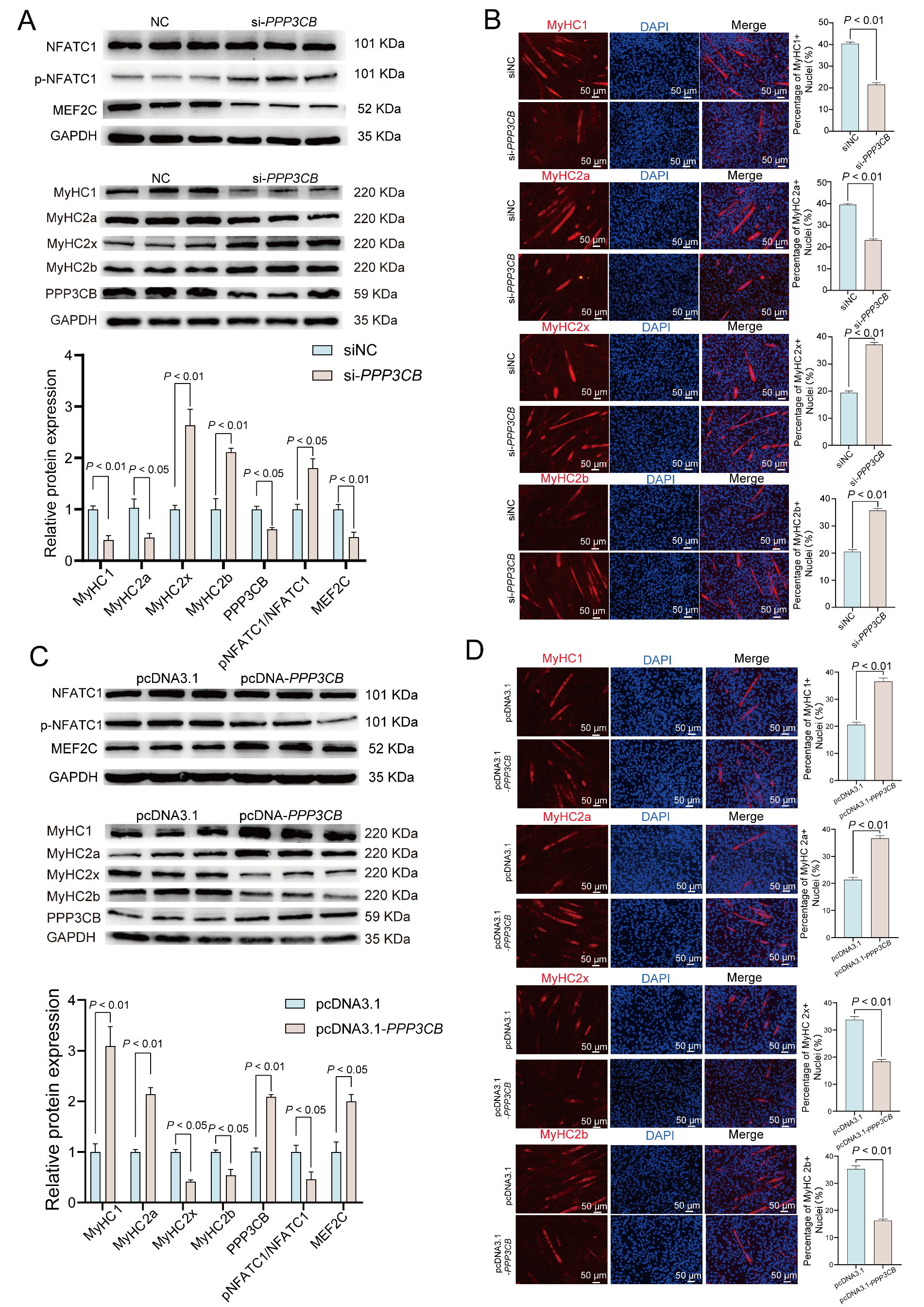


**Figure S16.** Summary of *PPP3CB* perturbation by knockdown and overexpression. A) Western blot analysis and densitometric quantification of fiber-type marker proteins and pathway-related proteins following *PPP3CB* knockdown (n = 3 per group). Band intensities were normalized to GAPDH. B) Quantification of the proportions of myotubes positive for distinct muscle fiber–type markers following *PPP3CB* knockdown (n = 3 per group). C) Western blot analysis and densitometric quantification of fiber-type marker proteins and pathway-related proteins following *PPP3CB* overexpression (n = 3 per group). Band intensities were normalized to GAPDH. D) Quantification of the proportions of myotubes positive for distinct muscle fiber–type markers following *PPP3CB* overexpression (n = 3 per group). Data are presented as mean ± SD; *P* values were calculated using an unpaired two-sided Student’s t-test.


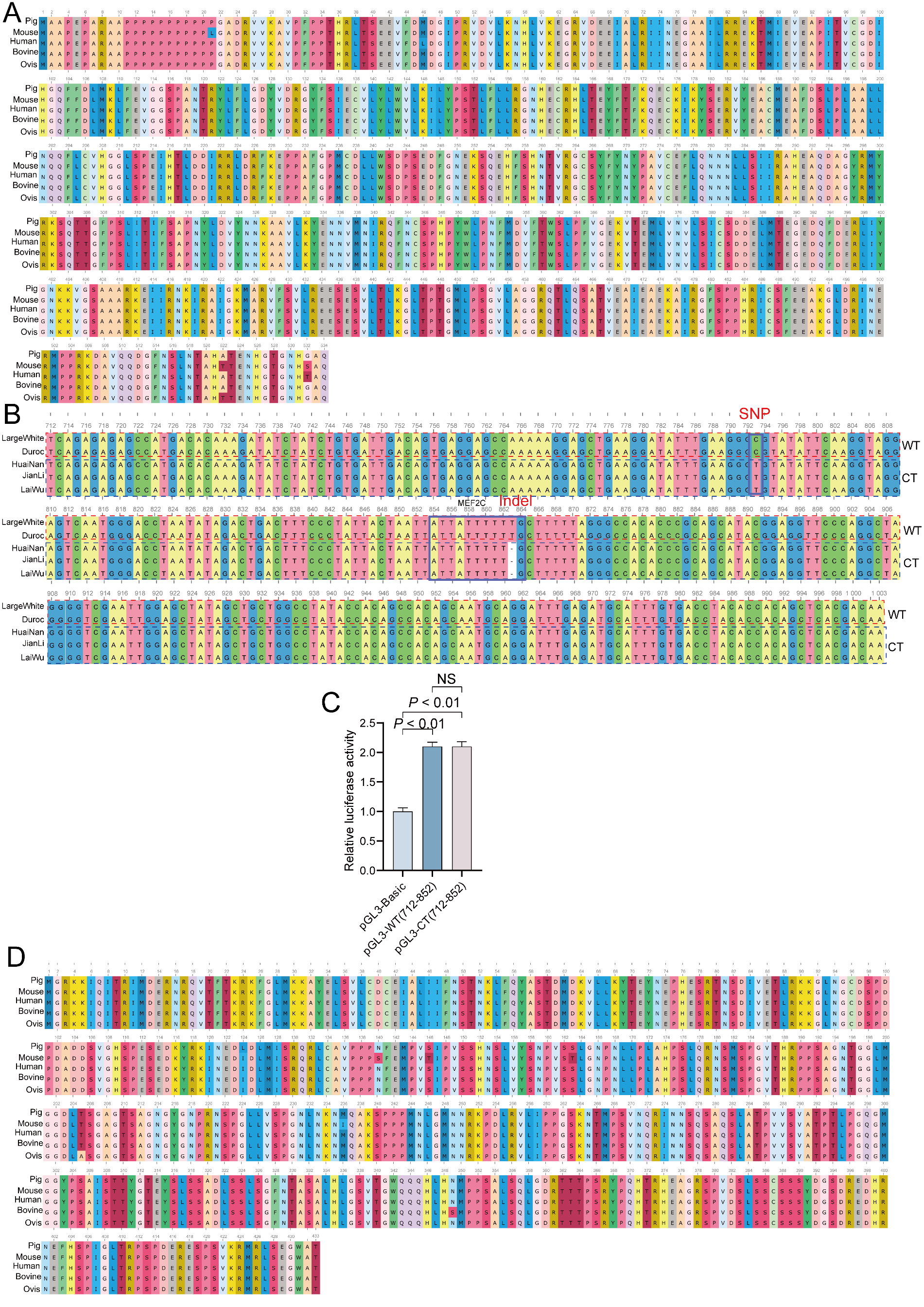


**Figure S17.** Analysis of amino acid sequence conservation of PPP3CB and MEF2C, and Analysis of *PPP3CB* promoter sequence in Chinese indigenous pigs and lean-type pigs. A) Conservation analysis of the PPP3CB amino acid sequence in pigs, mice, humans, bovines, and ovis. B) Analysis of the *PPP3CB* promoter sequence in Chinese indigenous pigs and lean-type pigs. C) Luciferase reporter assay of the *PPP3CB* promoter fragment (bp 712–852) harboring a SNP at position 793. D) Conservation analysis of the MEF2C amino acid sequence in pigs, mice, humans, bovines, and ovis.

Data are presented as mean ± SD; *P* values were calculated using an unpaired two-sided Student’s t-test.


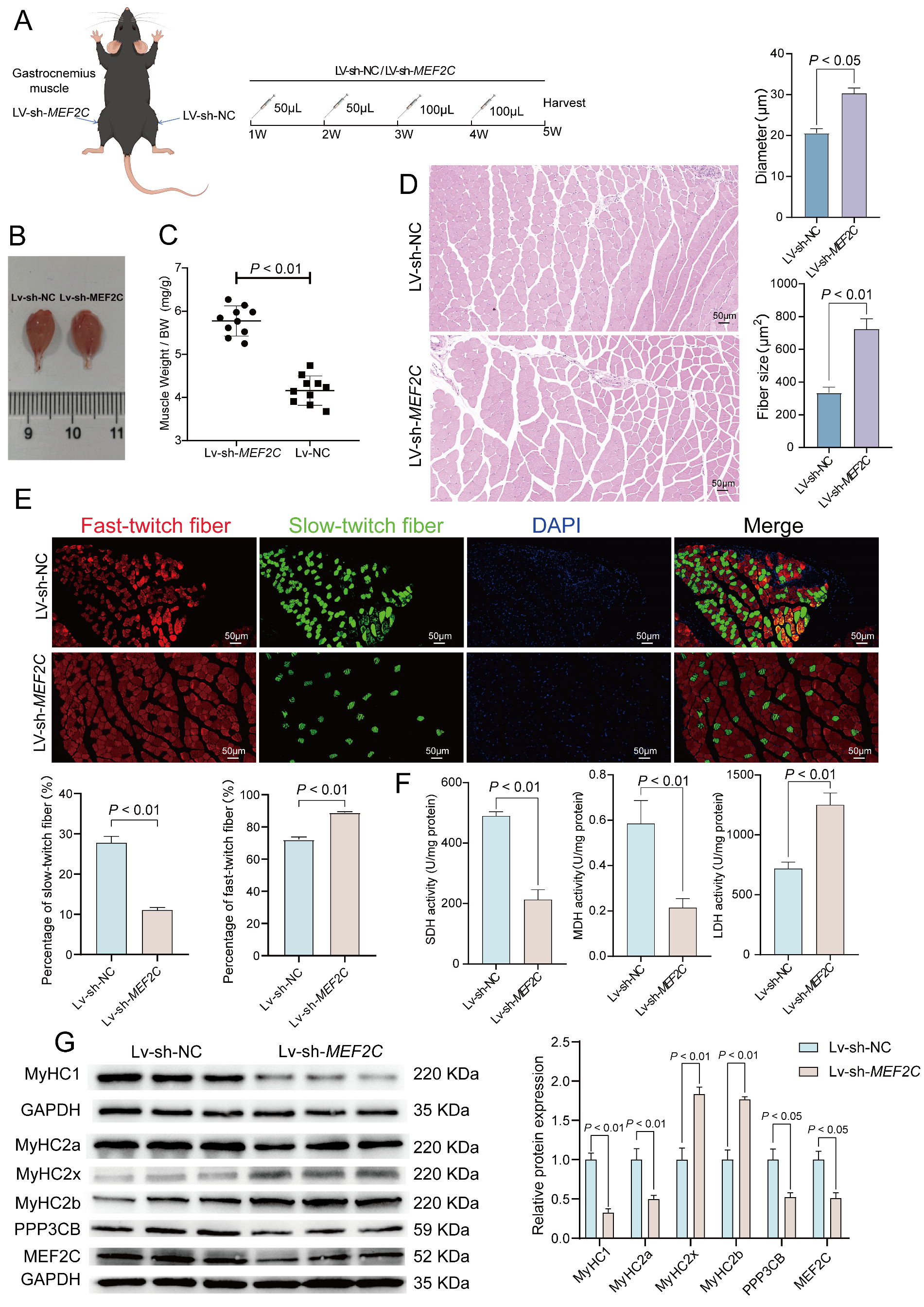


**Figure S18.** Lentivirus-mediated *MEF2C* knockdown in muscles significantly increased muscle mass and the proportion of glycolytic muscle fibers. A) Schematic of the in vivo injection procedure for lentivirus-mediated *MEF2C* knockdown. B) Representative gross morphology of gastrocnemius (Gas) muscles from control (LV-sh-NC) and *MEF2C* knockdown (LV-sh-*MEF2C*) mice. C) Quantification of ten independent experiments showed that lentivirus-mediated *MEF2C* knockdown significantly increased the weights of the Gas muscles (n = 10 for each group). *P* values were determined using a paired t-test. The data were normalized to body weight (BW) (mg/g). D) Representative H&E-stained sections of Gas muscles (n = 3 mice per group). For morphometric analysis, ≥150 myofibers were quantified per mouse, and per-mouse means were used for statistical analysis. Scale bar, 50 μm. E) Representative immunohistochemistry images of fast- and slow-twitch fiber types in Gas muscles from 2-month-old mice injected with LV-sh-NC or LV-sh-*MEF2C* (n = 3 mice per group). ≥150 myofibers were quantified per mouse, and per-mouse means were used for statistical analysis. Scale bar, 50 μm. F) Enzyme activities of SDH, MDH, and LDH in Gas muscles from 2-month-old mice injected with LV-sh-NC or LV-sh-*MEF2C* (n = 3 mice per group). G) Western blot analysis and densitometric quantification confirming *MEF2C* knockdown in Gas muscles (n = 3 mice per group). Band intensities were normalized to GAPDH. Data are presented as mean ± SD. *P* values were calculated using a paired two-sided Student’s t-test.


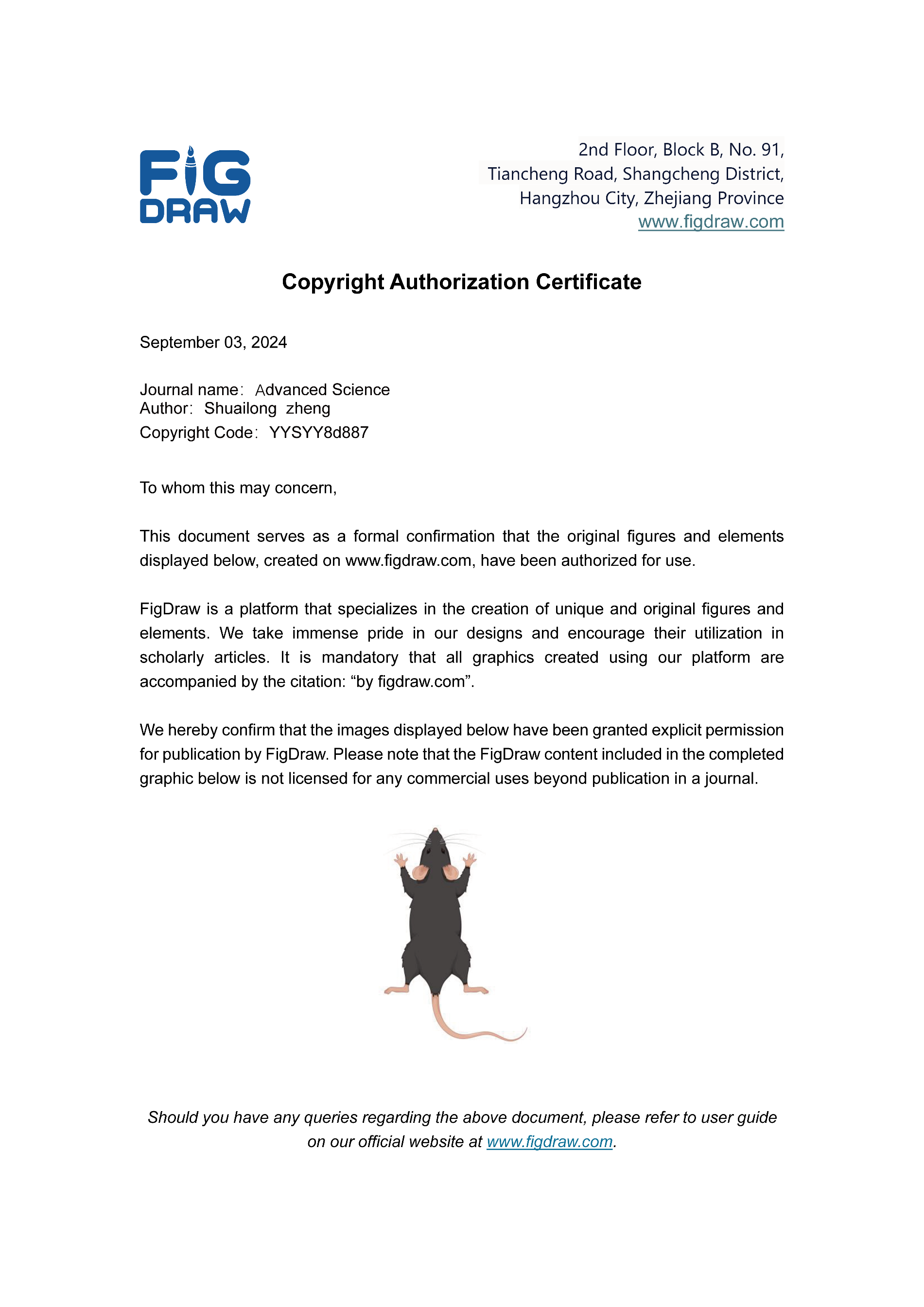


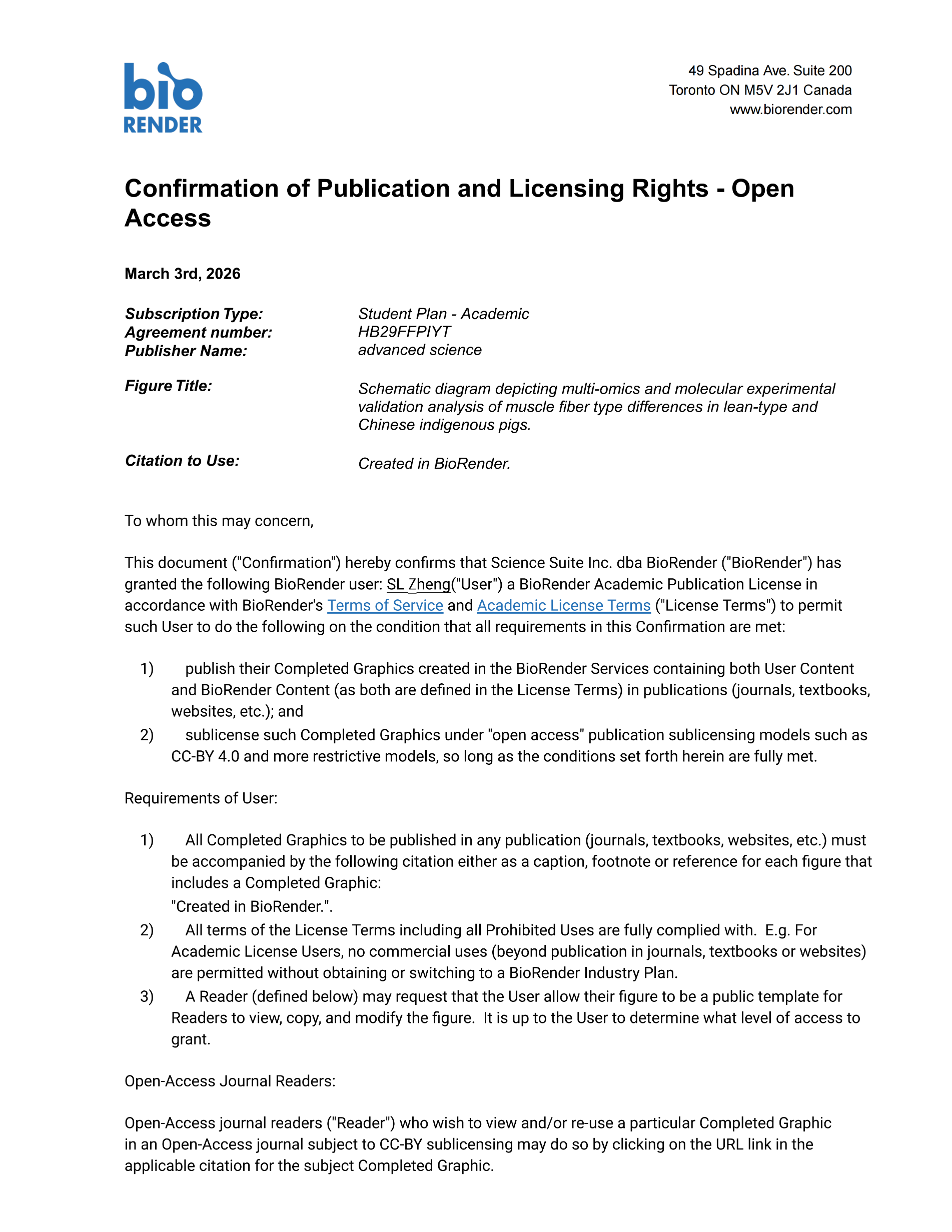


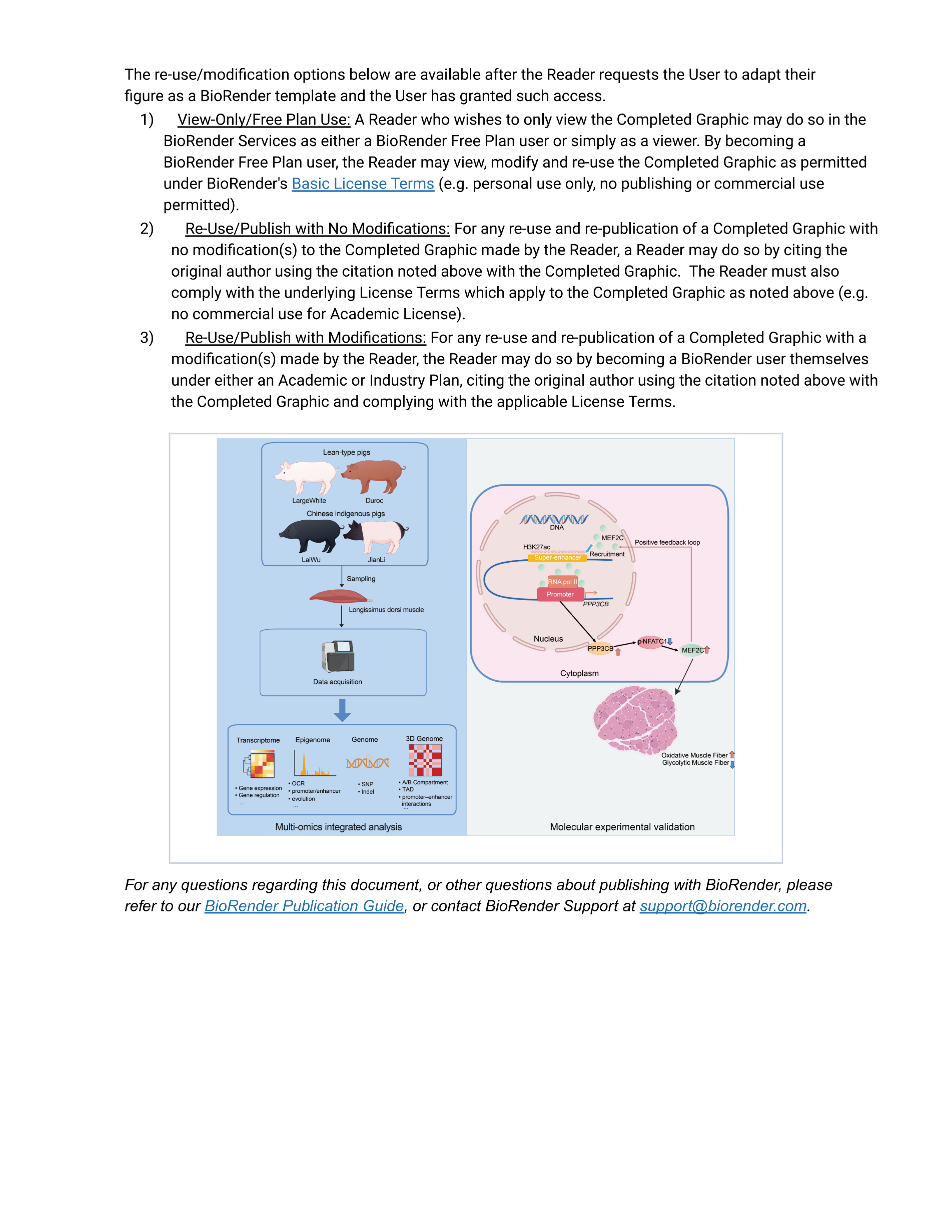

Supplement: Supplementary file 1 — Supporting File 1: advs75292‐sup‐0001‐SuppMat.docx. [file ADVS-13-e23959-s001.docx]
